# Oncogenic activation of SMYD3-SHCBP1 promotes breast cancer development and is coupled with resistance to immune therapy

**DOI:** 10.1038/s41419-025-07570-8

**Published:** 2025-03-29

**Authors:** Lihua Mo, Min Deng, Ragini Adhav, Yuni Chan, Josh Haipeng Lei, Sek Man Su, Xin Zhang, Tingting An, Jianlin Liu, Jianjie Li, Xiaodong Shu, Jun Xu, Yuqing Wang, Lin Chen, Yan-Gao Man, Ning-Yi Shao, Tingxiu Xiang, Chu-Xia Deng, Xiaoling Xu

**Affiliations:** 1 Cancer Centre, Faculty of Health Sciences, University of Macau, Macau SAR, China; 2 Centre for Precision Medicine Research and Training, Faculty of Health Sciences, University of Macau, Macau SAR, China; 3 Ministry of Education Frontiers Science Center for Precision Oncology, University of Macau, Taipa, Macau SAR, China; 4 Laboratory of Wound Repair and Rehabilitation Medicine, State Key Laboratory of Trauma, Burns and Combined Injury, Daping Hospital, Army Medical University, Chongqing, 400042 China; 5 Department of Pathology, Hackensack Meridian School of Medicine, Nutley, NJ USA; 6 Chongqing Key Laboratory of Translational Research for Cancer Metastasis and Individualized Treatment, Chongqing University Cancer Hospital, Chongqing, 400030 China; 7 Zhuhai UM Science & Technology Research Institute, Hengqin, China

**Keywords:** Breast cancer, Cancer therapy

## Abstract

Breast cancer initiation and progression are driven by various oncogenic factors and their effects on the surrounding microenvironments. Through integrative analysis of ChIP-sequencing and RNA-sequencing with fast proliferating mammary epithelial cells from pregnant Brca1^MKO^ and wild type (WT) mice, we found that elevated Smyd3-Shcbp1 signaling is featured with activation of the Ras-MAPK pathway and increased transcription activity in both premalignant mammary epithelium and tumor cells. Smyd3-Shcbp1 signaling shapes the tumor immunosuppressive microenvironment (TIME) and is associated with immune therapy resistance to PD1 antibody treatment. Trametinib, a potent inhibitor of MEK/MAPK, could reverse the expression of Smyd3 and Shcbp1 in both Brca1 mutant and WT tumor bearing mice. We further demonstrated that the combinatory treatment of trametinib together with PD1 antibody enhances the function of effector T cells, sensitizing tumors with elevated Smyd3 and Shcbp1 signaling to αPD1 treatment. This study advances the understanding of breast tumor progression and provides a new selective strategy for breast cancer patients.

## Introduction

The rate of occurrence for breast cancer is the highest among all cancers, with 2.26 million new cases and 68500 deaths in 2020 globally [1]. Over 90% of breast cancers occur sporadically. However, 5–10% of cases are inheritable and are associated with germline mutations in several cancer predisposition genes, mainly breast cancer-associated gene-1 and 2 (BRCA1/2) [2, 3]. In addition, approximately 0.2–3.8% of breast cancer cases are pregnancy-associated breast cancer (PABC) that occurs during pregnancy or within twelve months postpartum [4–6]. PABC has a more aggressive clinical course and higher mortality rate than other forms of breast malignancies [4, 7–10].

Many factors have been implicated in the formation of PABC, including family history and BRCA1/2 mutations, immune regulation, age at first pregnancy, style of breast feeding, breast involution, and levels of some signature genes, such as estrogen receptor alpha (ERα), transforming growth factor-beta (TGF-β), and matrix metalloproteinase (MMP) [6, 11–14]. However, while some studies have shown that PABC occurs more frequently in BRCA1 mutation carriers than in noncarriers [6, 11–13], other studies have indicated that it does not [15–17], Thus, the role and involvement of BRCA1 in PABC are complex and deserve further investigation.

BRCA1 is a well-known tumor suppressor that plays a critical role in many important biological processes, including DNA damage repair, the cell cycle, centrosome amplification, DNA replication, chromatin remodeling, protein ubiquitination, and the tumor microenvironment [18–23]. Studies also showed that BRCA1 deficient breast cancers are derived from ERα+ luminal epithelial cells and go through luminal-basal transformation during tumorigenesis [24–26]. Consistently, oophorectomy to minimize estrogen production in human BRCA1 mutation carriers and in mouse carrying mammary-specific BRCA1 mutations significantly reduced the frequency of breast/mammary cancer formation [27, 28], whereas the administration of tamoxifen, which blocks E2/ERα signaling, reduced cancer risk [29–31].

By analyzing *Brca1*^*Co/Co*^*;MMTV-Cre* (Brca1^MKO^) mice [32], we found previously that Brca1 deficiency resulted in the abnormal development of mammary gland at early stages of the pregnancy, characterized by reduced total number of mammary epithelial cells, increased apoptosis and higher fraction of BrdU+ cells in the early S-phase. This phenotype is accompanied by large-scale upregulation of gene expression, including many genes that encode replisomes [33]. Because Brca1 deficiency triggers cell proliferation defects [34], which could be rescued by activation and/or inactivation of some oncogenes and/ or tumor suppressors [35–38], we believe that the large-scale upregulated genes might facilitate Brca1-deficient cells to overcome lethality and be malignantly transformed. While this finding reveals the role of BRCA1 in maintaining genome stability during pregnancy, the downstream genes that bridge estrogen signaling and the large-scale upregulation of gene expression remain unclear. Such genes, if identified, could serve as therapeutic targets to suppress malignant transformation of premalignant cells and proliferation of cancer cells.

In this study, we specifically designed experiments in Brca1 mutant cells and mouse models to investigate these issues and identified SET and MYND domain-containing protein 3 (Smyd3) and Src homolog and collagen homolog binding protein 1 (Shcbp1) as a tumorigenic signaling that mediates actions of Brca1 and ERα for the development of PABC and none-PABC. We demonstrated that elevated Smyd3-Shcbp1 signaling could reprogram the tumor immunosuppressive microenvironment (TIME) and promote immune therapy resistance upon αPD1 treatment. Smyd3-Shcbp1 expression could be reversed by trametinib treatment, and the combinatory treatment of trametinib with αPD1 enhances the function of effector T cells and sensitizes mammary tumors with elevated SMYD3 and SHCBP1 to αPD1 treatment.

## Results

### Brca1-deficient mammary tissues exhibit abnormally increased transcriptional activity

**Fig. 1:**
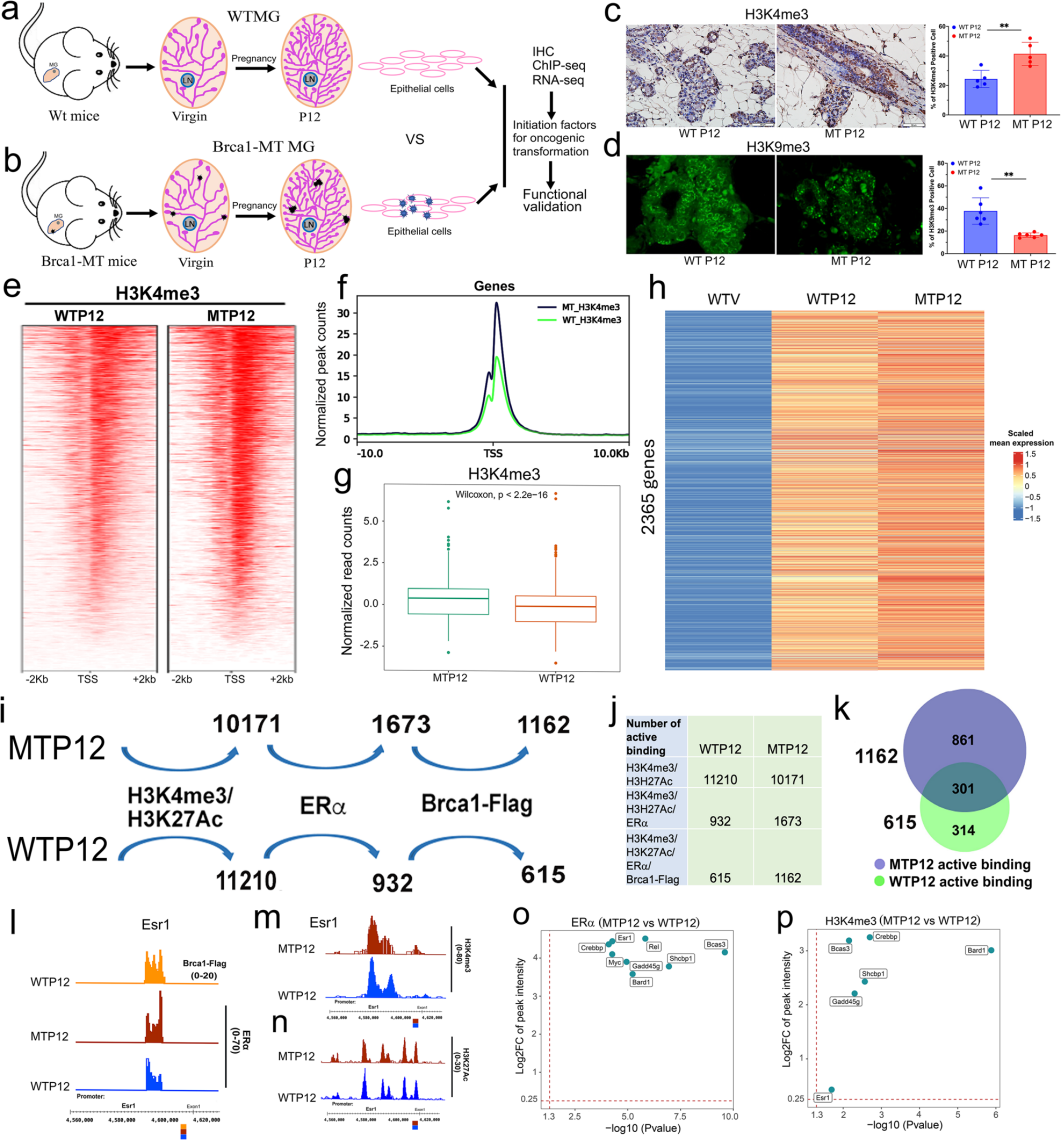
Abnormal transcription is associated with open chromatin from freshly isolated mammary epithelial cells in Brca1^MKO^ mice. Experiments designed for isolating fresh epithelial cells from mammary tissues of WTV, WTP12 (**a**), MTV, and MTP12 (**b**) mice. The triplicated samples (n = 6 mice/group) were collected from each genotype. The RNA-seq and ChIP-seq were performed and the candidate genes from combination analysis will be followed by functional analysis. Representative IHC images with antibodies of H3K4me3 (**c**) and IF staining with H3K9me3 (**d**) on WTP12 and MT P12 mouse mammary gland tissues and percent of positive cells from 5 images for H3K4me3 and 6 images for H3k9me3 were quantified by using ImageJ. (n = 3 mice/group). The binding intensity on promoter region across -2Kb and +2Kb of the TSS sites (**e**) and genome-wide (**f**) in both WTP12 and MTP12. MACS2 tools for peak calling with default parameters were used. By default, the q-value (minimum false discovery rate) cutoff for peak detection is set to 0.05. The plot was generated by the software “deeptools” (https://deeptools.readthedocs.io/en/develop/index.html. **g** ChIP-seq raw data were converted into the bam file and used the “ngsplot” tool to quantify and test the intensity for H3K4me3. **h** The upregulated genes in WTP12 and MTP12 with scaled mean expression from normalized counts of each gene are combined first with removed duplication gene names and then extract out the same gene list from WTV group to generate the heatmap by using the R package “pheatmap” with information in Supplementary data 3. Summary of analysis of genes based on overlapping binding patterns of Flag/ERα/H3K4me3/H3K27Ac (**i**), binding numbers with different combinations of antibodies (**j**), and distributions by Venn diagram 2.1 (**k**) in MTP12, WTP12, and WTP12/MTP12. The ChIP-seq data were calculated using the “clusterProfiler” R package with cut off values of p < 0.05 [77]. **l** Visualization of ChIP binding peaks of Esr1 gene against antibodies of Flag, (**l**, orange color), ERα antibody (burgundy color) in MTP12 cells and ERα antibody (blue color) in WTP12 cells. The images were generated using IGB (The Integrated Genome Browser free software for distribution and exploration of genome-scale datasets) [78]. **m** Visualization of ChIP binding peaks of Esr1 gene against antibody of H3K4me3 in MTP12 (burgundy color) and (blue color) in WTP12 cells. **n** Visualization of ChIP binding peaks of Esr1 gene against antibody of H3K27Ac in MTP12 (burgundy color) and (blue color) in WTP12 cells. Differential binding peak intensities binding analysis of Esr1, Bard1, Shcbp1, Myc, Rel, Bcas3, Gadd45g, and Crebbp by ERα (**o**) and Η3Κ4me3 (**p**) between MTP12 and WTP12 cells with diffReps version 1.55.4 [79]. Using likelihood-ratio test and annotated to the closest genes. Genes associated with at least one significant genomic region (P value < 0.05, that is, -log10(P value) > 1.3 and log2 (fold change) >0.25) were denoted as statistically differentially marked. When a gene is annotated with multiple significant genomic regions, the most significant one is assigned to that gene.

In the early pregnancy stage, mammary epithelial cells are forced to enter fast proliferating processes stimulated by ERα and may need strong transcriptional regulation and repair damage during DNA replication processes. To study impact of Brca1 deficiency on this, we isolated mammary epithelial cells of virgin (V) and pregnant day 12 (P12) mice at three-month old of age from both WT and Brca1^MKO^ mice [32] for immunohistochemistry (IHC), RNA-sequence and ChIP-sequence analyses (Fig. 1a, b). The mammary glands of Brca1^MKO^ mice and control mice at this stage were generally comparable in their alveologenesis (Supplementary Fig. 1a–c) and development from ductal numbers and markers (K14 and K18) analysis in majority of the mice (Supplementary Fig. 1d–g). Next, we performed the analysis of these samples, including WT virgin (WTV), mutant virgin (MTV), WT mice at pregnant day 12 (WTP12) and Brca1^MKO^ mice at pregnant day 12 (MTP12) (Supplementary Fig. 1h–m) with a focus on the expression changes between WTP12 and MTP12.

To examine the transcriptional activity of mammary tissues at P12, we stained the mammary tissues with antibodies of H3K4me3 and H3K9me3. The data showed much more H3K4me3-positive cells in MTP12 than in WTP12 glands (Fig. 1c). Consistently, fewer H3K9me3-positive cells were observed in MTP12 than in WTP12 glands (Fig. 1d). The binding intensity analysis of H3K4me3 on the promoter region between -2 kb and +2 kb also showed much higher in MTP12 with statistical significance (Fig. 1e–g). To identify genes and pathways with increased transcription or activities triggered by the open chromatin structure associated with Brca1 deficiency, next, we analyzed both RNA-seq and ChIP-seq. The data from bulk-RNA sequences (Supplementary data 1 and data 2) revealed a total of 2365 genes whose expressions are increased in both WTP12 and MTP12 mice compared to WTV mice, with a much greater increase in MTP12 mice (Fig. 1h and Supplementary data 3), indicating that more genes were upregulated MTP12 cells. To characterize these changes, we found that there were only six pathways enriched with significance in MTV vs WTV (Supplementary Fig. 1i). In MTP12 vs WTP12 (Supplementary Fig. 1j), there were 188 pathways enriched with significance, including positive regulation of ERK1 and ERK2, PI3K/AKT pathways and MAPK pathways, suggesting the activation of more tumor promoting factors in MTP12 due to Brca1 deficiency. For the pathways enriched by ERα, there are 10 common pathways were enriched in both WTP12 and MTP12 among the top 15 enriched pathways, 5 specific enriched pathways in WTP12 vs WTV, and 3 specific enriched pathways in MTP12 vs WTV (Supplementary Fig. 1k, l and Supplementary data 4), including “estrogen response early”, highlighting that Brca1 deficiency could induce Esr1 expression in mammary epithelial cells.

To narrow down the candidate genes, we performed ChIP-seq analysis. Because the lack of the reliable antibody to Brca1 for ChIP-seq, we first inserted a Flag sequence right into the endogenous *Brca1* gene before the stop codon to generate a Flag-Brca1 knocking mouse strain (Supplementary Fig. 2a–h). The homozygous mice for Flag-Brca1 (Flag/Flag) (Supplementary Fig. 2e–h) were phenotypically normal and were used as Brca1 WT mice for Flag ChIP assay. We then performed ChIP-seq with antibodies against Flag-Brca1 in WTP12 cells, H3K4me3, H3K27Ac, ERα in both Brca1 WTP12 and Brca1 MTP12 cells. The data revealed 6290 genes were pulled down by the Flag antibody in WTP12 cells, 2124 and 3684 genes by ERα in WTP12 and MTP12 cells, respectively (Supplementary Fig. 2i). The genes pulled down by H3K4me3 were 18,821 and 18620, whereas those pulled down by H3K27Ac were 14618 and 13763 in WTP12 and MTP12, respectively (Supplementary Fig. 2i and Supplementary data 5). Through the analysis, we detected 10171 genes and 11210 genes that bind to both H3K4me3 and H3K27Ac antibodies in MTP12 and WTP12, respectively, among which, 1673 and 932 bind to ERα antibody in MTP12 and WTP12, respectively, suggesting that Brca1 deficiency might more preferably increase the binding by ERα (Fig. 1i, j). Comparing these lists of genes with the 6290 genes pulled down by Flag-Brca1 in WTP12, there were 615 out of 932 genes in WTP12 and 1162 of 1673 genes in MTP12 that could bind to Brca1, respectively (Fig. 1i, j). When these numbers were merged, we detected 314 genes that appear only in WTP12, 861 genes appear only in MTP12, and 301 genes appear in both WTP12 and MTP12 (Fig. 1k and Supplementary data 6). To further investigate cancer-promoting effects, we performed KEGG pathway analysis of these three gene lists. The data revealed that the PI3K-AKT, MAPK, Ras oncogenic pathways were enrichened in 301 gene group but not other two gene lists (Supplementary Fig. 2j–l), showing that the genes in 301 gene group may contain the tumor promoting factors in MTP12 epithelial cells.

To validate the binding of the genes with these four antibodies, we examined 8 candidate genes that are involved in cancer progression, including Esr1 (Fig. 1l–n), Bcas3, and Bard1 (Supplementary Fig. 2m–p), as well as Crebbp, Myc, Rel, Gadd45g, and Shcbp1 (not shown). The data revealed that enhanced binding on Esr1, Bcas3, Bard1, and Shcbp1 locus with statistical significance by ERα, H3K4me3, H3K27Ac in MTP12 (Fig. 1o, p and Supplementary Fig. 2q). We concluded that loss function of Brca1 is associated with enhanced estrogen signaling and activation of the transcription of certain gene sets in MTP12 mammary epithelial cells.

### Smyd3 is upregulated in premalignant mammary tissues in Brca1^MKO^ mice and tumors

**Fig. 2:**
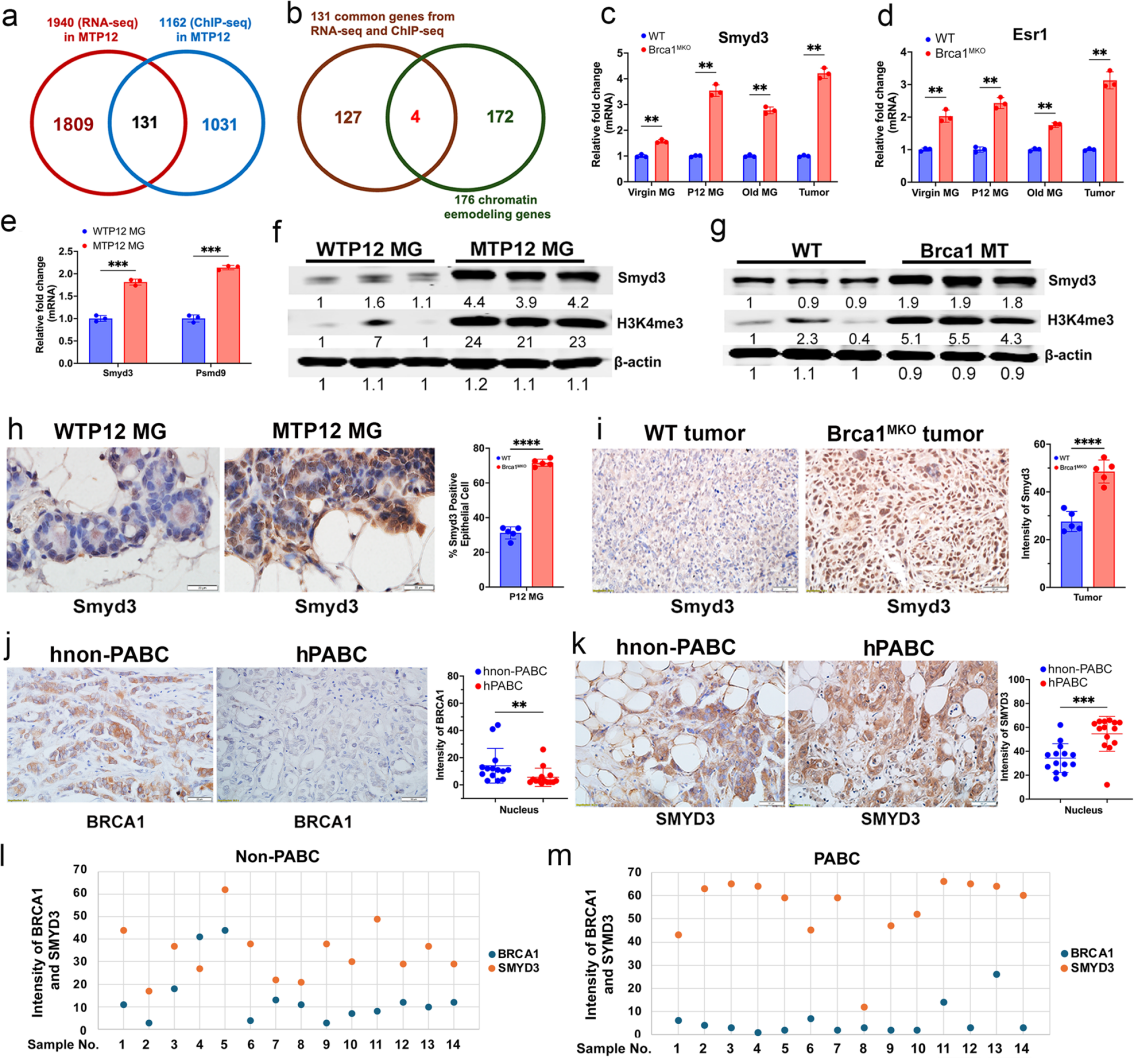
Smyd3 is upregulated in both Brca1 mutant mice and human breast cancer tissues. **a** 131 genes were obtained from comparisons of 1940 genes by RNA-seq in MTP12 mice to 1162 genes by ChIP-seq that could potentially be regulated by Brca1 in MTP12 mice. **b** Four genes, Esr1, Symd3, Hmgb1, and Terf1, were obtained by comparison of 131 genes with 176 chromatin remodeling genes which were obtained from existing gene set GO:0065004 in Supplementary data 7. **c**–**e** Expressions of Smyd3 (**c**), Esr1 (**d**) in virgin, P12, old mammary gland tissues, and tumors in both WT and Brca1^MKO^ mice. Expressions of Psmd9 (**e**) in both WTP12 and MTP12 MG tissues as determined by qPCR. (n = 3 mice/group). Protein levels of Smyd3, H3K4me3 in P12 MG (**f**) and tumors (**g**) in Brca1^MKO^ mice and their controls from p53+/− mice by Western blots. (n = 3 mice/group). The protein levels of Smyd3 in MG tissues from MTP12 and WTP12 mice (**h**), mammary tumors (**i**) by IHC and their quantifications. Five views were counted from each section. (n = 3 mice/group). Representative images of human breast cancer sections from non-PABC and PABC patients stained with antibodies of BRCA1 (**j**) and SMYD3 (**k**), and the quantifications of protein in the nucleus. A total of 14 non-PABC and 14 PABC were analyzed, and most samples are at the early invasive stage (Stage I or II) with both invasive ductal and lobular components. Protein levels of BRCA1 and SMYD3 in each non-PABC (**l**) and PABC (**m**) cancer patients visualized by dot plots.

To identify Brca1 target genes that are not only regulated by Brca1 but also involved in oncogenesis triggered by Brca1 deficiency, we compared the 1162 genes that were sequentially pulled down by antibodies of H3K4me3/H3k27Ac/ERα in MTP12 cells and also by Flag antibody in WTP12 cells (Fig. 1j), and the 1940 DEGs associated with the loss of Brca1 in MTP12 by RNA-seq. We found 131 shared genes that are bound by Brca1 in wild type cells and are also differentially expressed at P12 caused by Brca1 deficiency (Fig. 2a and Supplementary data 7). We sorted out genes that might be up regulated by Brca1 deficiency and compared the 131 genes to 176 chromatin remodeling-related genes from the existing GO:0065004 dataset that are enriched with protein‒DNA complexes (Supplementary data 7), and we obtained 4 genes, i.e. Smyd3, Esr1, Hmgb1 and Terf1 (Fig. 2b), of which, Smyd3 and Esr1 are among the top 20 candidate genes in Scatter plot (Supplementary Fig. 1m and Supplementary data 2) and selected for further investigation.

Smyd3 is a histone methyltransferase that specifically methylates Lys-4 of histone H3, and it induces di- and trimethylation (H3K4me2 and H3K4me3) but not monomethylation (H3K4me1) [39]. Esr1 is well known for its functions in enhancing mammary epithelial cell proliferation during pregnancy [40] and tumorigenesis [41] by affecting the expression of its downstream genes. While Hmgb1 and Terf1 may play many important biological functions, their connection to the induction of chromatin opening and large-scale gene upregulation is currently unclear. We therefore focused on the functions of Smyd3 and Esr1 in gene expression, tumor initiation and progression. We examined the expression of Smyd3, Esr1, and the genes downstream of Smyd3 by qPCR, IF staining, and the protein levels of Smyd3 and H3K4me3 by Western blot analysis in MTP12 mammary glands and tumors. The data revealed that the expression of Smyd3 and Esr1 at the mRNA level was increased in MTV, MTP12, and tumor tissues compared with age-matched Brca1-WT controls (Fig. 2c, d). The mRNA levels of Smyd3 downstream genes and genes involved in tumorigenesis, including Psmd9, Pik3cb, N-myc, CrkL, Wnt10b, Riz, Ccng1, Cdk2, and Map3k11, etc [42–44]. were all increased in premalignant MTP12 mammary tissues and tumor tissues (Fig. 2e and Supplementary Fig. 3a–d). The protein levels of Smyd3 and H3K4me3 were also elevated in both MTP12 and tumor tissues (Fig. 2f, g). Furthermore, Smyd3 protein levels in MTP12 mammary glands and tumor tissues of Brca1^MKO^ mice were elevated compared to their controls, as shown by IHC staining (Fig. 2h, i). These data suggest that the elevated expression of Smyd3 and Esr1 could serve as potential oncogenic initiators prior to tumor formation.

Because Smyd3 was elevated in both P12 premalignant mammary epithelial cells and malignant tumor tissues of Brca1^MKO^ mice, we then explored whether elevated SMYD3 levels could affect cancers diagnosed in human PABC. We performed IHC with antibodies against BRCA1 and SMYD3 in PABC and non-PABC samples. The data showed that non-PABC has higher SMYD3 protein level than the normal control mammary tissues (Supplemental Fig. 3e–i), PABC patients, in general, exhibited lower protein levels of BRCA1 (Fig. 2j), but higher protein levels of SMYD3 (Fig. 2k) compared to the non-PABC patients and such a difference was also observed at individual level (Fig. 2l, m). These data suggest that SMYD3 might play a role in promoting mammary tumor growth in these patients. To explore whether Smyd3 could play a role in breast cancer development in general, we analyzed SMYD3 expression in breast cancers from cBioportal datasets, and the data showed that approximately 24% of breast cancer samples had amplification of Smyd3 gene (Supplementary Fig. 3j, k) and correlated with high expression of SMYD3 (Supplementary Fig. 3l, m). Further analysis of different types of breast cancer (Supplementary Fig. 3n, o) showed that approximately 30% of invasive lobular carcinomas exhibited SMYD3 amplification (Supplementary Fig. 3p), and high expression of SMYD3 was also observed in invasive lobular carcinoma and invasive ductal carcinoma in the Curtis and TCGA breast cancer database (Supplementary Fig. 3q, r). Altogether, these data provide a strong correlation between elevated levels of SMYD3 in PABC, BRCA1, and invasive lobular breast cancers, making it a very important therapeutic target to interrogate in breast cancer.

### ERα and Smyd3 signals enhance mammary tumor progression in Brca1-deficient mice

**Fig. 3:**
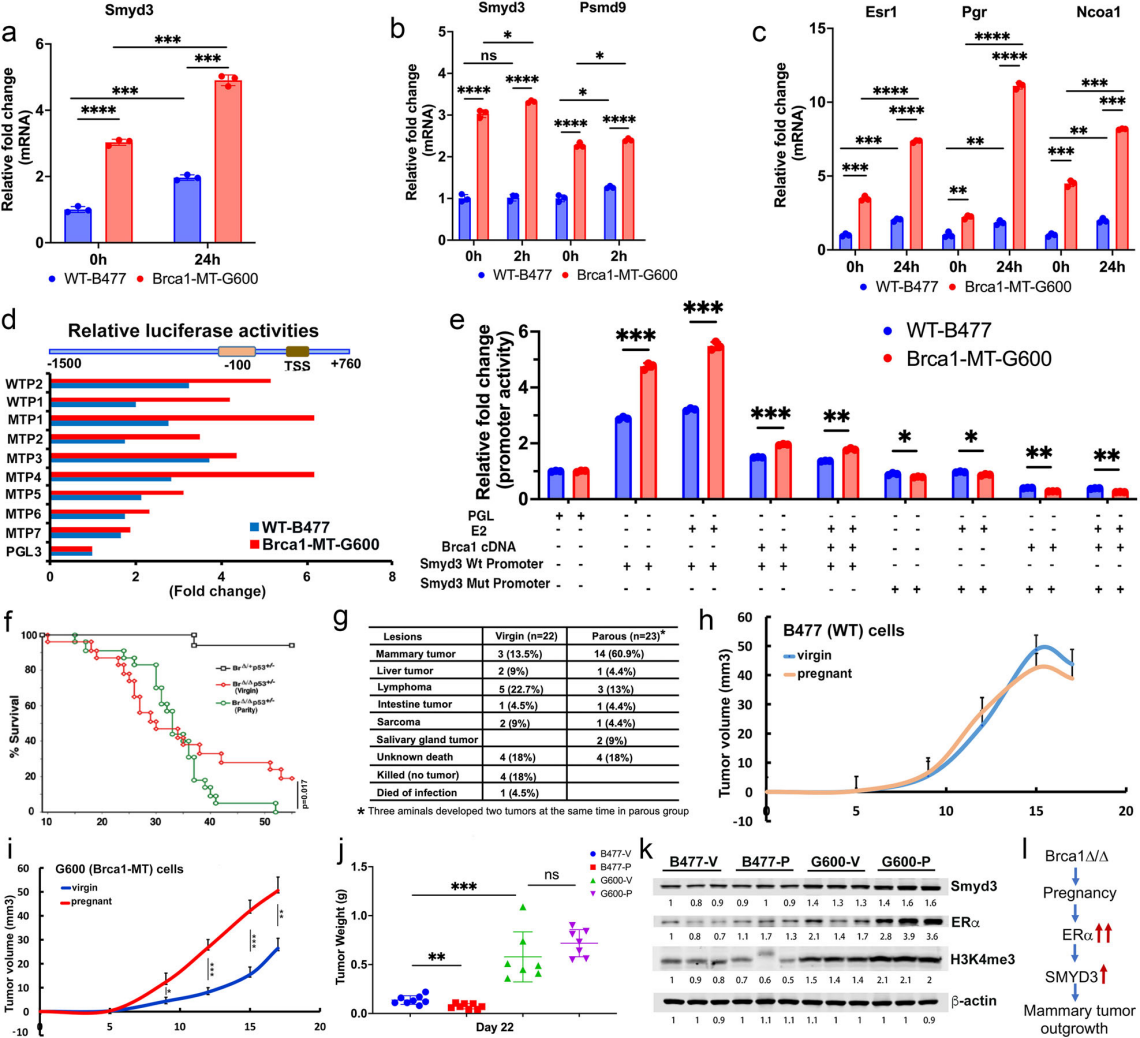
Smyd3 is negatively regulated by Brca1 but positively regulated by ERα in mammary tumor initiation and progression. **a** The expression pattern of Smyd3 in WT and Brca1 MT mammary epithelial cell lines at 0 and 24 hours after 100 nM estradiol (E2) treatment as determined by qPCR. **b** The expression of Smyd3, and Psmd9 under E2 treatment for 2 hours in WT-B477 (WT) and Brca1-MT G600 (MT) cells. **c** The Expression of Esr1, Pgr, and Ncoa1 after 24 hours of 100 nM E2 treatment as determined by qPCR in B477 (WT Brca1) and G600 (Brca1-MT) cell lines. **d** Luciferase promotor activity assay from different fragments of Smyd3 promoters, including the fragment from -1500 bps to + 760 bps (WTP2), -1160 bps to +760 bps serve as (WTP1), -800 bps to +760 bps (MTP1), -600 bps to + 760 bps (MTP2), -400 bps to +760 bps (MTP3), -250 bps to +760 bps (MTP4), -100 bps to +760 bps (MTP5), -50 bps to +760 bps (MTP6), and -1160 bps to -288 bps (MTP7). **e** Luciferase activity assay using WTP1 and MTP7 of the Smyd3 promoter under E2 treatment or overexpression of mBrca1 cDNA in both WT B477 and Brca1 MT G600 mammary epithelial cell lines. **f** Kaplan-Meier tumor-free curve of Brca1^Δ11/Δ11^;p53^+/−^ under virgin (n = 22) or parous (n = 23) conditions. Virgin Brca1^Δ11/+^;p53^+/−^ mice (n = 15 mice) were also set up as controls and only one of them developed lymphoma at about 7 months of age. All animals were kept under the same house condition. The parous mice had gone through two full-term of pregnancies. The survival free mice starting from 35 weeks between Brca1 mutant virgin and parous mice is significantly different by Fisher’s exact test (p = 0.017). **g** Comparison of mammary tumor incidence after experiencing two times pregnancies (14/23, 60%) vs virgin (3/22, 13.5%) in Brca1^Δ11/Δ11^;p53^+/−^ mice by Fisher’s exact test (p = 0.002). **h** The tumor growth curve of nude mice with implantation of WT B477 cells in both virgin and pregnant groups (n = 8 mice/group). **i** The tumor growth curve of nude mice with implantation of MT G600 cells in both virgin and pregnant groups (n = 7 mice/group). **j** The plot of tumor weight at Day 22 after fat pad implantation in nude mice of B477 and G600 cells in both virgin groups and groups with experience of pregnancy from the same cohort of mice in **h** and **i**. **k** The protein levels of Smyd3, ERα, and H3K4me3 in tumor tissues of the same cohort of mice in **j**. **l** Summary of a signaling pathway from Brca1 deficient mice.

Since our data revealed that Smyd3 and ERα signaling is elevated in both MTP12 cells and breast cancer tissues, we hypothesized that the actions of cancerous factors or pathways that contribute to tumorigenesis and progression could be stimulated by elevated ERα and Smyd3 signaling in rapidly proliferating MTP12 mammary epithelial cells during early pregnancy in mice. To test this hypothesis, we examined whether the expressions of Smyd3 and its downstream genes could be induced by 17β-estradiol (E2) treatment or inhibited by tamoxifen in both WT (B477) and Brca1 mutant (G600) cell lines. We found that Smyd3 expression was already higher in Brca1 mutant cells and could be further increased by E2 treatment (Fig. 3a), as well as Smyd3 downstream gene Psmd9 (Fig. 3b and Supplementary Fig. 4a), and estrogen-responsive genes, including Esr1, Pgr, and Ncoa1 (Fig. 3c and Supplementary Fig. 4b). Of note, the induced expression of Esr1, Smyd3, and Psmd9 by E2 could be suppressed by tamoxifen treatment in G600 cells (Supplementary Fig. 4c–e).

**Fig. 4:**
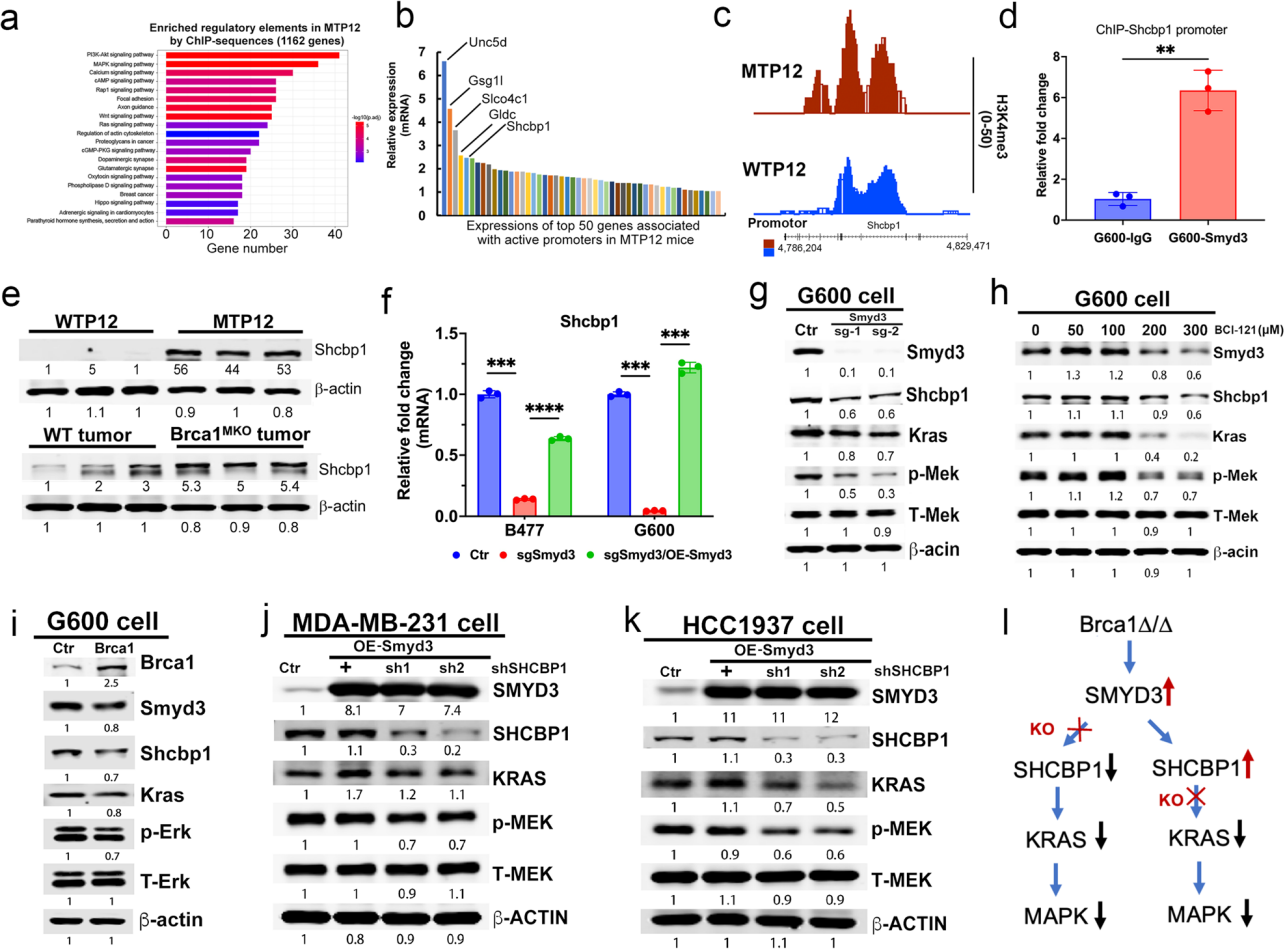
Smyd3 potentiates Shcbp1 and oncogenic Kras-MAPK pathways. **a** Bar charts showing the top 20 enriched KEGG pathways from the 1162 genes identified in MTP12 by ChIP-seq analysis which were shown in Fig. 1i–k. **b** The rank of the top 50 common genes based on upregulated genes (1940) from MTP12 vs WTV by RNA-seq and 1162 genes with active transcription activities from MTP12 by ChIP sequence analysis. **c** The Shcbp1 binding patterns of mammary epithelial cells in both WTP12 and MTP12 mice with antibodies of H3K4me3 generated from Integrated Genome Browser (IGB). **d** The enrichment binding of the Shcbp1 promoter by Smyd3 antibody and IgG by ChIP-qPCR in Brca1-MT cells. **e** Shcbp1 protein levels in mammary tissues from WTP12, MTP12, tumors from WT and Brca1^MKO^ mice (n = 3 mice/group) by Western blots. **f** The Shcbp1 expression in both B477 and G600 cells expressing sgSmyd3 and sgSmyd3/OE-Smyd3 as determined by qPCR. **g** The Protein levels of Smyd3, Shcbp1, pMek, and Kras in G600 cells expressing sgSmyd3 by Western blots. **h** Protein levels of Smyd3, Shcbp1, Kras, and pMek in Brca1-MT cells treated with the Smyd3 protein inhibitor BCI-121 at different concentrations, as shown by Western blots. **i** The protein levels of Brca1, Smyd3, Shcbp1, Kras, and pErk in G600 cells with OE-mBrca1 cDNA as shown by Western blots. The protein levels of SMYD3, SHCBP1, KRAS and pMEK in OE-SMYD3-231 (**j**) or OE-SMYD3-HCC1937 cells (**k**) with the expression of shSHCBP1 as shown by Western blots. **l** Summary for Brca1-Smyd3-Shcbp1 signaling regulation and oncogenic pathways.

To investigate whether Smyd3 expression was directly regulated at the transcriptional level by ERα and Brca1, the Smyd3 promoter and the promoters with serial deletions were cloned and inserted into the pGL3 luciferase reporter vector (Supplementary Fig. 4f). the luciferase activity assays revealed that 1) the promoter activities were greatly reduced if the fragment from -250 to the TSS site (MTP5) was deleted (Fig. 3d), suggesting that this fragment is a key region for transcriptional regulation. 2) The promoter activity was higher in G600 cells than in B477 cells and much higher in G600 cells after E2 treatment (Fig. 3e). 3) The promoter activities were suppressed in both WT and Brca1-MT cells if Brca1 cDNAs were introduced into both WT and MT mammary epithelial cells (Fig. 3e), and 4) the promoter activities were still suppressed after E2 treatment when Brca1 cDNA was expressed in both WT and MT cells (Fig. 3e). These data demonstrated that the Smyd3 promoter not only are positively regulated by ERα but also negatively regulated by Brca1 and the effect of ERα on the Smyd3 promoter could be overridden by Brca1.

Next, we investigated whether the pregnancy could affect mammary tumor development in mice carrying the Brca1 mutation. In the Brca1^MKO^ mice, the tumorigenesis is caused by MMTV-Cre mediated deletion of Brca1 in the mammary epithelial cells, which is enhanced by pregnancy [32], therefore, the Brca1^MKO^ mice are not an ideal model for studying this. Thus, to directly compare the impact of pregnancy on Brca1 mediated tumorigenesis, we used a strain of mice that carries a germline deletion of the full-length Brca1 (Δ11), which causes lethality at middle gestation but the mutant mice can survive to adulthood in a p53 + /− mutation background (*Brca1*^*Δ11/Δ11*^*;p53*^*+/−*^) [35]. We divided the *Brca1*^*Δ11/Δ11*^*;p53*^*+/−*^ mice into two groups, with one group undergoing two full cycles of pregnancy before 4 months of age (parous group) and the other group maintained as virgin mice. The data revealed that both groups of mice had similar cancer-free survival in the first 35 weeks and that tumorigenesis was slowed down in the virgin group after 35 weeks (Fig. 3f). However, when only mammary tumors were counted, we found that approximately 60% of mice (14/23) in parous group developed mammary tumors, whereas 13.5% of virgin mice (3/22) developed mammary tumors during the same period (Fig. 3g). Otherwise, a comparable number of *Brca1*^*Δ11/Δ11*^*p53*^*+/−*^ mice (10/22 in the virgin group and 8/23 in the parous group) also developed other types of cancers, including liver cancer, lymphoma, intestinal cancer, sarcoma, and salivary gland cancer (Fig. 3g).

To further explore whether mammary tumor growth with Brca1 deficiency could be affected by ERα, we performed allograft experiments. First, we prepared two groups of nude mice, i.e., virgin mice and mice that were mated to induce pregnancy. Then, we implanted either WT B477 cells or Brca1 mutant G600 cells into the fat pads of these mice at the day when we detected vaginal plug was detected (0.5 day of pregnancy, P0.5) and monitored tumor growth. The tumors formed by WT B477 cells did not have an obvious difference in their growth for the first 17 days (Fig. 3h). In contrast, Brca1 mutant G600 cells grew much faster in the mice with pregnancy than in virgin mice during the same period (Fig. 3i), although this effect became less obvious when tumors were well established in later days (Fig. 3j). The data also showed that the protein levels of ERα, Smyd3, and H3K4me3 in tumors generated by Brca1 mutant G600 cells were higher than tumors generated by WT B477 mice, and the pregnancy further enhanced their expression as revealed by both Western blot (Fig. 3k) and IHC staining (Supplementary Fig. 4g–n) analyses. Altogether, these findings showed that elevated ERα signaling could enhance Smyd3 expression and cell proliferation in vitro and tumor growth in pregnant mice with G600 Brca1 mutant cells (Fig. 3l).

### Smyd3 potentiates Kras/MAPK signaling through the oncogenic action of Shcbp1

Our data indicated that the expression of Smyd3 could be stimulated by ERα in synergy with the loss of Brca1 and enhance tumor outgrowth in nude mice with Brca1 mutant cells. We wanted to further understand the factors or pathways that could mediate the oncogenic actions of ERα and Smyd3. Thus, we conducted KEGG pathway analysis with 1162 genes that had combined binding of H3K4me3, ERα, and Flag-Brca1, and the data revealed that the five well-known oncogenic pathways, including the cancer, PI3K-AKT, Ras, MAPK, and Rap1 pathways, were among the top 10 enriched pathways (Fig. 4a and Supplementary data 8). Further comparative analysis using the genes upregulated in MTP12 obtained by RNA-seq (1940) (Supplementary data 2) and ChIP-seq (1162) (Supplementary data 5) identified 131 common genes, including Unc5d, Gsg1l, Slco4c1, Gldc, and Shcbp1, that were highly expressed in the MTP12 mammary gland (Fig. 4b). Our analysis of these top candidates indicated that the expression levels of Shcbp1 were not only frequently upregulated in various cancers, including breast cancer [45], but were also identified in several signature gene lists of breast cancers, including 4 genes [46, 47], 5 genes [48], 8 genes [49], 9 genes [50], and 12 genes [51], for predicting survival, metastasis, and drug responses. We observed more binding on the promoter region of Shcbp1 by H3K4me3 in P12 Brca1^MKO^ mice by IGB and binding peak intensity analysis (Fig. 4c, and Fig. 1p) and our ChIP‒qPCR results also demonstrated that the binding of Smyd3 to the Shcbp1 promoter was enriched more than 5-fold in Brca1 mutant G600 cells compared to IgG controls (Fig. 4d). The positive correlation was also observed between the expression of Smyd3 and Shcbp1 in premalignant virgin MG, P12 mammary and tumor tissues at both protein and mRNA levels (Fig. 2c, f, g, Fig. 4e and Supplementary Fig. 5a), indicating Shcbp1 might mediate Smyd3 signaling. Therefore, we decided to focus on the action of SHCBP1.

SHCBP1 is reported to activate MAPK/ERK/MEK through Shc, Grb2, Sos, and Kras signaling pathways in cancer cells [52, 53]. We found that the expression levels of all these genes were elevated in the mammary gland and tumor tissues of WT and Brca1^MKO^ mice (Supplementary Fig. 5b, c). We next conducted a functional analysis to examine Shcbp1 expression by introducing sgSmyd3 or sgSmyd3/OE-Smyd3 in both WT-B477 and Brca1 mutant G600 cells (Supplementary Fig. 5d, g). The data revealed that cells with sgSmyd3 in both cell lines had reduced expression levels of Shcbp1 (Fig. 4f) and all these 4 genes, in addition to Smyd3 downstream gene Psmd9 (Supplementary Fig. 5e–i). Conversely, the reduced gene expression could be restored when Smyd3 cDNA was introduced back to both the G600 (Fig. 4f and Supplementary Fig. 5e, f) and B477 (Supplementary Fig. 5h, i) cells expressing sgSmyd3. These data suggest that Smyd3 acts upstream of Shcbp1 to regulate expression of these genes.

We showed earlier that MAPK and ERK pathway were enriched in the MTP12 vs WTP12 enrichment pathway analysis. To examine whether aberrant transcriptional regulation by Smyd3 could affect the protein levels of Shcbp1 and Kras-MAPK signals, we overexpressed (OE) two different sgRNAs of Smyd3 in Brca1 mutant G600 cells, and found that the protein levels of Shcbp1, Kras, and pMek were decreased while the total Mek was unchanged (Fig. 4g). Similar data were obtained with the treatment of BCI-121, a Smyd3 inhibitor (Fig. 4h). Conversely, reconstitution of Brca1 by introducing Brca1 cDNA in G600 cells repressed the Shcbp1-MAPK signaling (Fig. 4i), demonstrating that Smyd3 acts at the upper level of the Shcbp1 and Kras-MAPK oncogenic pathways. In human MDA-MB-231 cells (BRCA1-WT) and HCC1937 cells (BRCA1-MT), expression of shSHCBP1 reduced KRAS-MAPK oncogenic signals in both cell lines (Fig. 4j, k). These data demonstrate that the Kras-MAPK pathway could be activated by elevated Smyd3-Shcbp1 levels in Brca1 deficient cells and minimized by ablation of Smyd3 or Shcbp1, respectively (Fig. 4l).

### Silencing of SMYD3 or SHCBP1 impairs the oncogenic actions of the Kras-MAPK pathway and inhibits mammary tumor growth

**Fig. 5:**
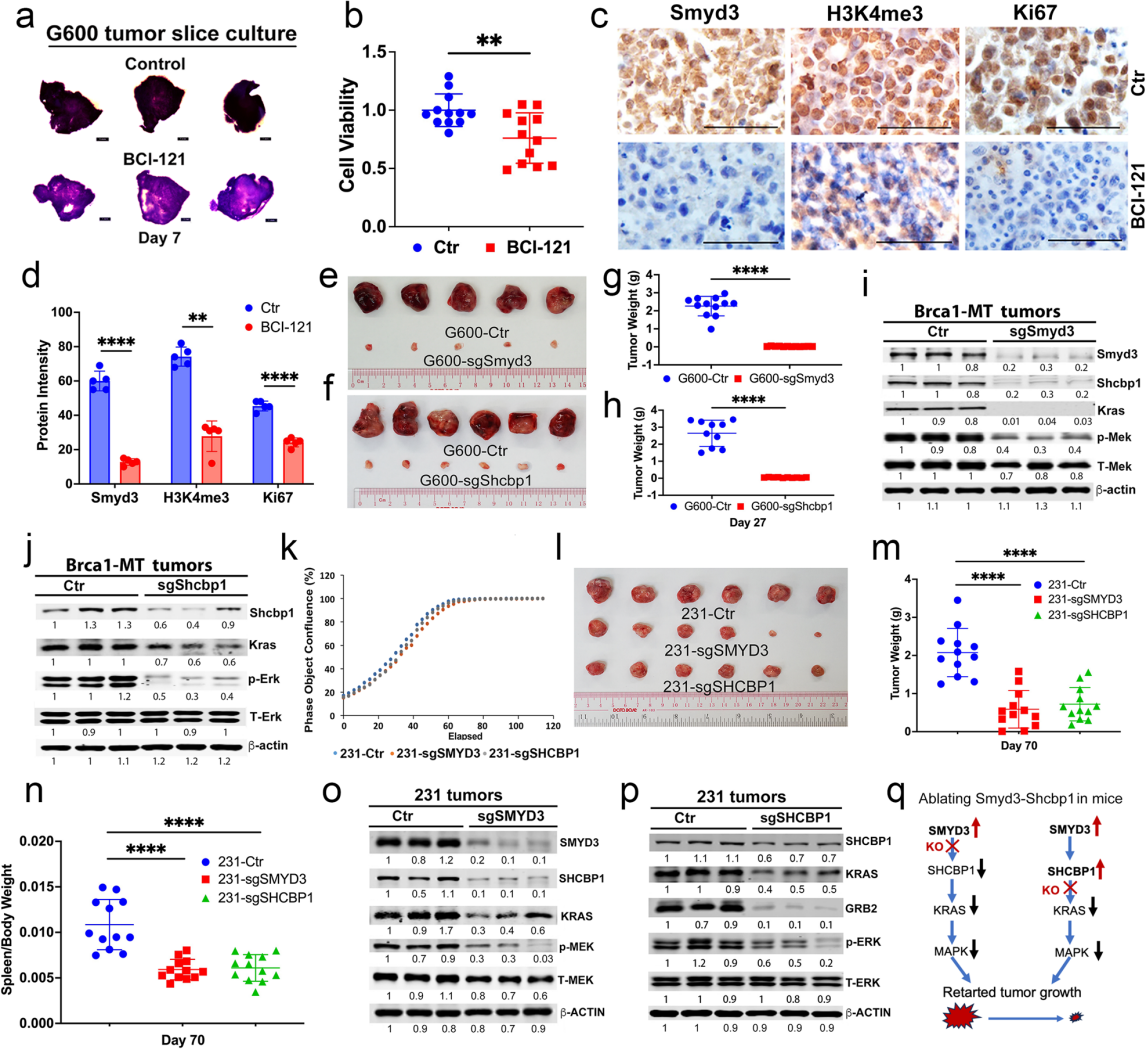
Disruption of SMYD3 and SHCBP1 decreases the activities of oncogenic pathways. **a**, **b** Representative images of tumor slices from G600 tumors without (control) or with BCI-121 treatment at a concentration of 300 µM for 7 days (**a**). The MTT assay was performed after harvesting the slices 7 days later and the quantifications of cell viability (**b**) in (**a**) (n = 3 mice/group). The IHC against antibodies of Smyd3, H3K4me3, Ki67 (**c**) and quantifications of protein intensities (**d**) from the same cohort of tumor slices in (**a**, **b**) (n = 3 mice/group). Scale bar is 50 μm. Representative tumor images from nude mice implanted with G600 parental and sgSmyd3-G600 (**e**) and sgShcbp1-G600 (**f**) in mammary fat pads with 2 × 10^5^ cells per mouse for 27 days (n = 10-12 mice/group). The tumor weight plots from the same cohort of mice with expression of sgSmyd3 (**g**) or sgShcbp1 (**h**). The protein levels of Smyd3, Shcbp1, Kras, pMek, and pErk in tumors initiated with parental G600, sgSmyd3-G600 (**i**), and sgShcbp1-G600 cells (**j**) in nude mice as shown by Western blots. **k** The cell growth curve of MDA-MB-231 (231) parental, sgSMYD3-231, and sgSHCBP1-231 cells was measured by IncuCyte. Tumor images of parental 231, sgSMYD3-231, and sgSHCBP1-231 cells (**l**) and plot of tumor weight (**m**) in nude mice (n = 12 mice/group). **n** The plot of relative spleen weight from the nude mice with the implantation of parental MDA-MB-231 (231), sgSMYD3-231, and sgSHCBP1-231 cells at 2 × 10^6^ cells per mammary fat pad for 70 days (n = 12 mice/group). The protein levels of SMYD3, SHCBP1, KRAS, GRB2, pMEK, and pERK in tumors initiated with parental 231, sgSMYD3 (**o**), and sgSHCBP1 cells (**p**) in nude mice by Western blots. **q** Summary of tumor growth after disrupting either Smyd3 or Shcbp1 in mice.

Our above functional regulation studies showed that elevated levels of Smyd3 act as an upstream factor of Shcbp1 in vitro. We then wanted to explore whether an inhibitor of Smyd3, BCI-121 [54], could inhibit its oncogenic action in vivo, however the data indicated that BCI-121 did not elicit strong effect in suppressing tumors with implantations of G600 and 231 cell under our treatment condition. We then used a semi-in vivo 3-dimentional tumor slice culture (3D-TLC) platform [55] to examine whether BCI-121 could inhibit Smyd3 action in tumor tissue sections. We sliced freshly dissected tumor tissues and cultured them in a 24-well plate under treatment with BCI-121 for 7 days. We found that cell death was significantly increased after BCI-121 treatment compared to the controls, as revealed by MTT assay (Fig. 5a, b and Supplementary Fig. 6a, b). IHC staining with antibodies against Smyd3, H3K4me3, and Ki67 revealed that the protein levels of Smyd3, H3K4me3, and Ki67 were dramatically decreased in tumor slices treated with BCI-121 (Fig. 5c, d and Supplementary Fig. 6c, d), suggesting that the inhibition of Smyd3 could disable the downstream oncogenic actions potentiated by Smyd3 and ERα.

To further confirm whether the inhibitory effect is indeed due to the inhibition of Smyd3 or Shcbp1, we orthotopically implanted G600 cells stably expressing either sgSmyd3 or sgShcbp1 into the fat pad of nude mice and monitored tumor growth. The data showed that

either sgSmyd3 or sgShcbp1 in these cells did not affect cell growth in vitro (Supplementary Fig. 6e), however, it did significantly inhibit tumor growth in vivo (Fig. 5e–h). Consistently, the protein levels of Shcbp1, Kras, and pMek were significantly decreased in tumor tissues expressing either sgSmyd3 or sgShcbp1 compared to the parental control tissues (Fig. 5i, j). It is known that tumor growth in mice often causes splenomegaly phenotype due to immune dysregulation and accumulation of abnormal immune cells [56]. We also found that tumorigenesis associated with G600 cells resulted in splenomegaly, which was reversed by the disruption of either Smyd3 or Shcbp1 (Supplementary Fig. 6f, g). These findings demonstrate that Smyd3 and Shcbp1 are dominant oncogenic drivers and that silencing Smyd3 and Shcbp1 in epithelial cells could minimize tumor growth and block their negative effect on abnormal immune responses in spleen tissues in mice.

Next, we conducted further tests in immune-competent mice by using 545 cell line which was derived from mammary tumors of Brca1^MKO^ mice and could grow in FVB mice. We observed a similar anticancer effect of sgSmyd3 or sgShcbp1 (Supplementary Fig. 6h, i). In addition, we orthotopically implanted human MDA-MB-231 and HCC1937 cells stably expressing either sgSMYD3 or sgSHCBP1 into the fat pad of nude mice and monitored tumor growth. The data revealed that disruption of either SMYD3 or SHCBP1 in MDA-MB-231 cells did not affect cell growth in vitro (Fig. 5k) but significantly inhibited tumor growth in both 231 and HCC1937 mice models (Fig. 5l, m and Supplementary Fig. 6k, l) with reversed the splenomegaly phenotype (Fig. 5n and Supplementary Fig. 6j). Consistent with the profound anticancer effects, the protein levels of SMYD3, SHCBP1, KRAS, and pMEK were decreased in the tumors with either sgSMYD3 or sgSHCBP1 expression compared to the parental tumors (Fig. 5o, p). These data demonstrate that breast tumor progression could be minimized when the functions of either SMYD3 or SHCBP1 are disrupted (Fig. 5q).

### Elevated Smyd3-Shcbp1 signaling shapes the immunosuppressive microenvironment in mammary tumors

**Fig. 6:**
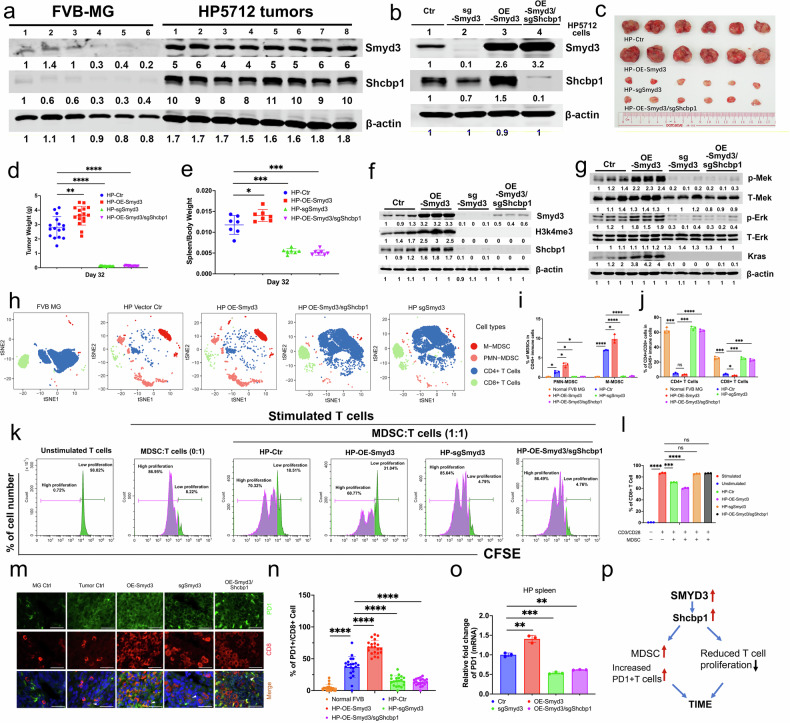
Elevated levels of SMYD3 and SHCBP1 promote the accumulation of MDSCs and reduce T-cell populations in mammary tissues. **a** The protein levels of Smyd3 and Shcbp1 in normal mouse mammary glands, and tumors initiated with HP5712 cells in 3 months old FVB virgin mice as shown by Western blots. **b** The protein levels of Smyd3 and Shcbp1 in HP5712 cells expressing sgSmyd3, OE-Smyd3, or OE-Smyd3/sgShcbp1 by Western blots. Representative tumor images (**c**) and tumor weight plots (**d**) at day 32 from FVB virgin mice implanted with HP5712 parental (HP tumors), OE-Smyd3-HP5712, sgSmyd3-HP5712, and OE-Smyd3/sgShcbp1-HP5712 cells at 1X10^6^ cells per mammary fat pad (n = 8 mice/group). **e** The plot of relative spleen weight from the same cohort of mice in **c** and **d**. **f** The protein levels of Smyd3, H3K4me3, and Shcbp1 in tumors initiated with HP5712 parental, OE-Smyd3-HP5712, sgSmyd3-HP5712, and OE-Smyd3/sgShcbp1-HP5712 cells by Western blots. **g** The protein levels of pMek, pErk, and Kras in tumors initiated with HP5712 parental, OE-Smyd3-HP5712, sgSmyd3-HP5712, and OE-Smyd3/sgShcbp1-HP5712 cells as shown by Western blots. **h** tSNE visualized immune cells from normal FVB mouse mammary glands (FVB MG, n = 6 mice), tumors implanted with parental HP5712 cells (HP Vector Ctr), HP-OE-Smyd3, HP-OE-Smyd3/sgShcbp1, and HP-sgSmyd3 from FVB mice (n = 6 mice/group) by CyTOF analysis. Quantifications of PMN-MDSCs and M-MDSCs (**i**), CD4+ and CD8 + T cells (**j**) in CD45+ immune cell populations from the same cohorts of mice in **h** (n = 3 biological independent samples/per group). **k**, **l** The WT-T cells were activated with CD3 and CD28 antibody. Co-cultured the activated T cells without or with MDSCs from the mice implanted with HP parental cells, HP cells expressing Smyd3, or sgSmyd3, or OE-Smyd3/sgShcbp1 for 72 hours and examined T cells proliferation status (**k**) and quantifications (**l**) of data in **k** (n = 3 mice/group). Representative images of CD8+/PD1+ double positive cells in mammary tissues from the mice with the genotypes of HP-Ctr, HP-OE-Smyd3, HP-sgSmyd3, and HP-OE-Smyd3/sgShcbp1 (**m**) and quantifications of CD8+/PD1 double positive cells from mammary tissues (**n**) in these mice (n = 4 mice/group, and at least 20 images for each sample were counted). Scale bar: 20 μm. arrow heads point to PD1/CD8 positive cell. **o** Expressions of PD1 from the spleen with implantation of parental HP cells, HP cells with OE-Smyd3, HP-sgSmyd3, and HP-OE-Smyd3/sgShcbp1 in FVB mice as determined by qPCR. **p** Summary of Smyd3-Shcbp1oncogenic signals shape the TIME.

The tumor immunosuppressive microenvironment (TIME) plays a very important role in supporting cancer outgrowth and metastasis [57], yet the effect of SMYD3-SHCBP1 signaling on the TIME is currently unclear. Given that the disruption of either Smyd3 or Shcbp1 could inhibit the tumor outgrowth, we hypothesized that SMYD3-SHCBP1 signaling might shape TIME benefiting tumor growth. To test this hypothesis, we used the HP (HP5712) cell line, which was derived from a spontaneous mammary tumor in an FVB female mouse [58] with relatively high protein levels of both Smyd3 and Shcbp1 (Fig. 6a). We first performed functional analysis of HP cells overexpressing (OE) of Smyd3, sgSmyd3, or OE-Smyd3/sgShcbp1 (Fig. 6b) in tumor formation and data revealed that tumor growth was markedly increased by the implantation of HP-OE-Smyd3 cells compared to the HP parental cells, whereas the tumor growth was dramatically decreased in the tumors expressing sgSmyd3 or OE-Smyd3/sgShcbp1 with reduced splenomegaly (Fig. 6c–e and Supplementary Fig. 7a, b). In support of this notion, elevated protein levels of Smyd3, H3K4me3, and Shcbp1 (Fig. 6f), and p-Mek, and p-Erk and Kras (Fig. 6g) were detected in tumor tissues with OE-Smyd3, but these protein levels were significantly decreased in tumor tissues expressing sgSmyd3 or OE-Smyd3/sgShcbp1. These data were consistent with a previous finding that SMYD3 could activate MAP kinase signaling through methylating MAP3K2 and promote the formation of Ras-driven carcinomas [59] and remodel the tumor microenvironment by activation of MEK/ERK [60]. Next, we examined the immune cell populations in the mammary glands of WT FVB mice and tumor tissues implanted with parental HP cells, OE-Smyd3-HP cells, OE-Smyd3/sgShcbp1-HP cells, or sgSmyd3-HP cells by CyTOF analysis with antibodies against CD8 and CD4 for T cells and Cd11b, Ly6G, and Ly6C for MDSCs. The data revealed that the effector cell populations, including CD4+ and CD8+ cells, were decreased in tumors with HP parental and HP-OE-Smyd3 cells compared to mammary tissues of control mice (Fig. 6h–j) but increased significantly in tumors with HP-OE-Smyd3/sgShcbp1 or HP-sgSmyd3 cells (Fig. 6h–j). In contrast, the protector cell populations, including both M-MDSCs and PMN-MDSCs, were increased in FVB mice with HP parental and HP-OE-Smyd3 tumors (Fig. 6h–j) and were decreased in tumors with HP-OE-Smyd3/sgShcbp1 and HP-sgSmyd3 cells.

To further examine whether T cell proliferations are due to the inhibition of MDSCs in these mice with HP tumors, we cocultured 2-month-old WT T cells with MDSCs from the mice with HP parental cells, HP cells expressing OE-Smyd3, or sgSmyd3, or OE-Smyd3/sgShcbp1. The data revealed that the proliferation of T cells decreased from 86.95% to 70.32% when they were cocultured with MDSCs from the tumor tissues with HP parental cells and further decreased to 60.77% when cocultured with MDSCs from HP cells with OE-Smyd3. T-cell proliferation was recovered when T cells were cocultured with MDSCs from tumors expressing either sgSmyd3 or OE-Smyd3/sgShcbp1 (Fig. 6k, l), demonstrating the strong inhibition of T cell proliferation by MDSCs in tumor tissues with elevated Smyd3-Shcbp1 oncogenic signaling. Because both CyTOF and coculture experiments showed that T cell proliferation was inhibited and that this inhibition could be attributed to immune checkpoint blockade mediated by the PD-1/PD-L1 interaction [61, 62], we next examined the protein levels of PD1 in T cells by co-staining with antibodies against CD8 and PD1 in these tumor tissues. The data revealed increased amount of CD8 and PD1 double-positive cells in mammary tumor tissues implanted by HP cells with elevated expression of Smyd3, but this increase was suppressed in tumor tissues with the expressions of sgSmyd3 or OE-Smyd3/sgShcbp1 (Fig. 6m, n) and PD1 expression level in the spleen of mice implanted with OE-Smyd3 cells were increased but were largely suppressed in mice implanted with sgSmyd3 or OE-Smyd3/sgShcbp1 cells (Fig. 6o). Because Smyd3 was identified form premalignant MTP12 cell, we also tested whether pregnancy could shape the TME and benefit the tumor growth with implantation of HP cells at P15 and 7 days after birth. The data revealed that the tumors in the pregnant group were two times bigger in volume than those in the virgin group (Supplementary Fig. 7c–e). Examining the immune cell component at these two time points revealed that MDSC populations were increased significantly at both P15 and 7 days after birth (Supplementary Fig. 7f, g). The CD8 + T-cell population was decreased significantly on these days (Supplementary Fig. 7h, i) compared to that in virgin mice, suggesting that elevated Smyd3-Shcbp1 oncogenic signaling could enhance the TIME during pregnancy and short period of time after birth in FVB mice.

Consistent with our study in mice, TCGA data analysis also showed a negative correlation between SMYD3 and activated CD8 cell population (Supplementary Fig. 7j) and a positive correlation between SHCBP1 and the MDSC population (Supplementary Fig. 7k). These data demonstrate that Smyd3-Shcbp1 oncogenic signaling could reduce the function of effector cells and shape the TIME in mammary tumor tissues of both pregnant and non-pregnant mice (Fig. 6p).

### Combinatory treatment with trametinib and αPD1 reverses Smyd3-Shcbp1 expression in tumor progression

Our data demonstrated that OE-Smyd3 activates MEK/ERK signaling (Fig. 6g), and it was reported that the protein level of SMYD3 is sensitive to the combination treatment of trametinib (Tra, which inhibits MEK1 and MEK2) and navitoclax in PDX (patient-derived xenografts) for non-small cell lung cancer [63]. Since Tra is a MEK inhibitor and has been approved by the FDA as an anticancer drug for melanoma, we next studied the effect of Tra on mammary tumor formation in mice. We implanted G600 cells into the fat pad of nude mice and treated the mice with Tra one week later. The data revealed that tumor growth was severely retarded in the Tra treatment group with decreased protein levels of pMek, pErk, Kras, and Grb2 (Supplementary Fig. 8a–c). Additionally, the splenomegaly phenotype was reversed (Supplementary Fig. 8d, e), without obvious cytotoxicity in the spleen and liver (Supplementary Fig. 8f), demonstrating that Tra has a significant inhibitory effect in mammary tumors with elevated Smyd3-Shcbp1 signaling.

**Fig. 7:**
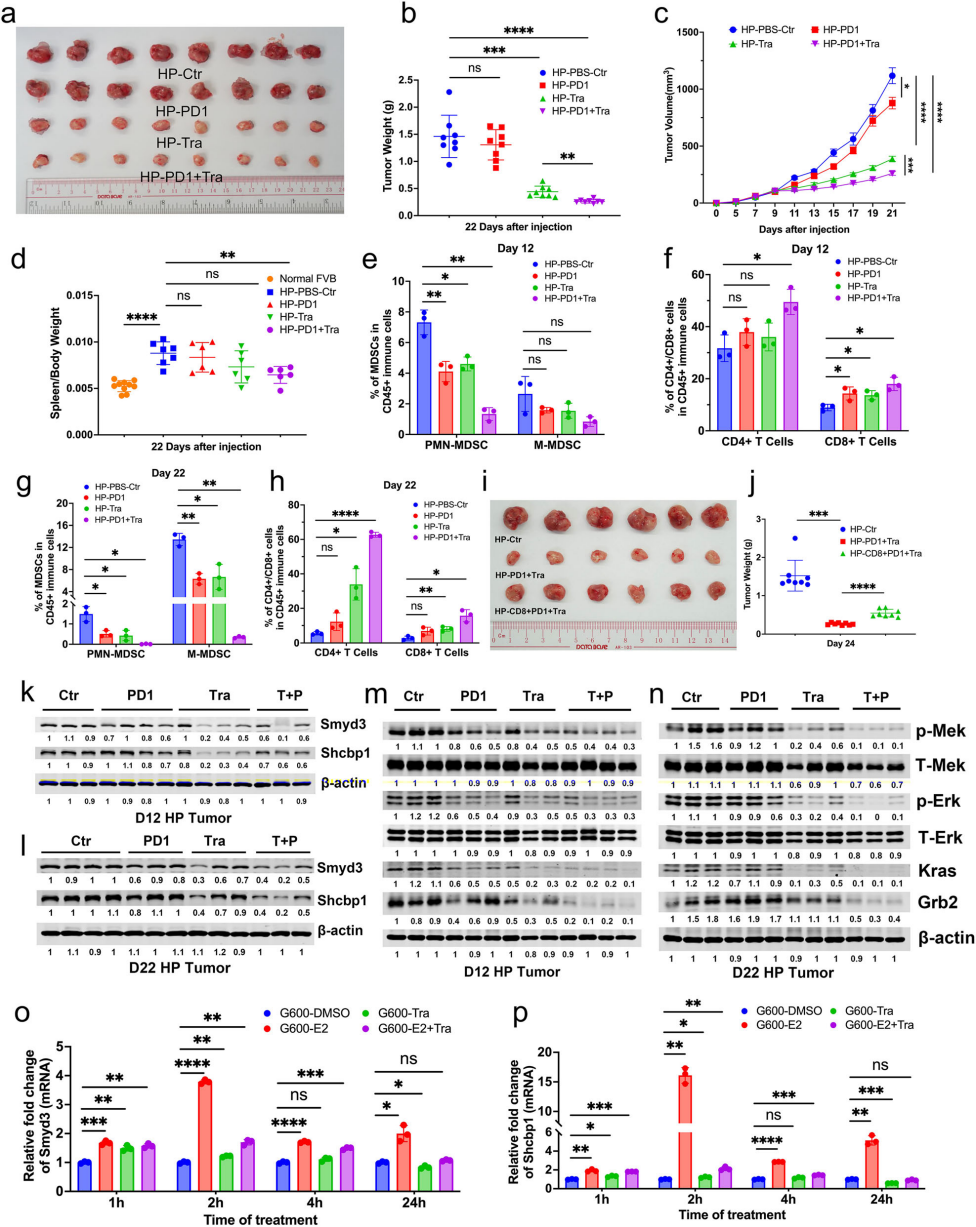
Combination treatment with αPD1 and trametinib reduced mammary tumor progression and increased the effector T-cell population. Representative tumor images (**a**), and tumor weight plots (**b**) at day 22 from FVB mice implanted with HP5712 cells at 1×10^6^ cells per mammary fat pad, and treatment with PBS, PD1, trametinib (Tra), and PD1+Tra (n = 8 mice/group). **c** The plot of tumor volume in the processes of treatment in (**a**, **b**). **d** The plot of relative spleen weight from the same cohort of mice in **a**, **b**. Quantifications of PMN-MDSCs and M-MDSCs (**e**), CD4+ and CD8 + T cells (**f**) in CD45+ immune cell populations from the same cohorts of mice in **a**, **b** by CyTOF analysis at D12 (n = 3 mice/group). Quantifications of PMN-MDSCs and M-MDSCs (**g**), CD4+ and CD8 + T cells (**h**) in CD45+ immune cell populations from the same cohorts of mice in **a**, **b** by CyTOF analysis at D22 (n = 3 mice/group). **i**, **j** Tumor images from the HP Ctr mice, HP mice treated with αPD1 and Tra, and HP mice treated with αPD1 and Tra with depletion of T cell using CD8 antibody (**i**). The plot of tumor weight (**j**) in the same cohort of mice in (**i**) (n = 8 mice in each group). The protein levels of Smyd3 and Shcbp1 in tumors initiated with HP5712 cells and treatment with PBS, αPD1, Tra, and αPD1+Tra in FVB mice at D12 (**k**) and D22 (**l**) as shown by Western blots. The protein levels of pMek, pErk, Kras, and Grb2 in tumors initiated with HP5712 cells and treatment with PBS, αPD1, Tra, and αPD1+Tra in FVB mice at D12 (**m**) and D22 (**n**) as shown by Western blots. Expressions of Smyd3 (**o**) and Shcbp1 (**p**) in G600 cells with the treatment of E2, Tra, and E2 together with Tra at 1 hour, 2 hours, 4 hours, and 24 hours as determined by qPCR.

Because elevated Smyd3-Shcbp1 signaling increases the PD1 and CD8 double-positive cell population (Fig. 6n), we next explored single and double treatment with Tra and αPD1 in tumor-bearing FVB mice, which have an intact immune system, with implantation of HP5712 cells. The data revealed that compared to the parental control tissues, αPD1 had no obvious effect. However, the combinatory treatment of αPD1 and Tra elicited a much stronger effect on tumor weight and volume (Fig. 7a–c) with a reversed splenomegaly phenotype (Fig. 7d and Supplementary Fig. 8g). In support of this notion, CyTOF analysis revealed that MDSCs started to decrease at day 12 and continued to decrease until day 22 (Fig. 7e, g and Supplementary Fig. 8h). Moreover, CD4+ and CD8+ cells started to increase (Fig. 7f, h and Supplementary Fig. 8h) in the Tra group, and these effects were enhanced in the double treatment group. To investigate whether the effects of Tra and αPD1 could be mediated by T cells, we depleted CD8 T cells in mice implanted with OE-Smyd3 tumor cells under the combination treatment of Tra and αPD1. The data revealed that the depletion of CD8 T cells in this mouse model reversed the effects of αPD1 and Tra (Fig. 7i, j), showing that T cells could mediate, at least in part, the killing effect in mice with elevated Smyd3-Shcbp1 under the combination treatment of Tra and αPD1.

Intriguingly, the protein and mRNA levels of Smyd3 and Shcbp1 began to decrease in both the Tra single treatment and double treatment groups on day 12 (Fig. 7k and Supplementary Fig. 8i), and the effects were much stronger on day 22 (Fig. 7l and Supplementary Fig. 8j). These results show that treatment with Tra may reverse the expression of Smyd3 and Shcbp1, which could serve as a good therapeutic drug for breast cancer patients with high levels of Smyd3-Shcbp1 signaling. Consistent with this, the protein levels of pMek, pErk, Kras, and Grb2 were also decreased significantly in both the Tra single and double treatment groups on both day 12 (Fig. 7m) and day 22 (Fig. 7n) without obvious toxicity in the liver and spleen (Supplementary Fig. 8m, n). Tra is an FDA-approved drug for the treatment of patients with V600E-mutated metastatic melanoma. Thus, we explored whether treatment with this drug could reverse the expression of Smyd3/Shcbp1 induced by estrogen (E2) since loss of Brca1 inhibition could elevate Smyd3 expression in both G600 and HP cell lines. The data revealed that E2 induces both Smyd3 and Shcbp1 expression at multiple time points, and the expression of these two genes could be repressed by Tra at 24 hours in both groups treated with Tra only or E2 and Tra together (Fig. 7o, p and Supplementary Fig. 8k, l). These results demonstrate that the combination treatment of Tra and αPD1 sensitizes mammary tumors with high levels of Smyd3 and Shcbp1 to αPD1 treatment by reversing the oncogenic expression of Smyd3 and Shcbp1 and increasing the functional T-cell populations in mice in general.

## Discussion

In this study, we showed that BRCA1 deficiency together with ERα signaling enhances SMYD3-SHCBP1 expression, which activates their downstream signaling, leading to the formation of mammary tumors in mice. BRCA1 is induced in the early stages of pregnancy, which antagonizes the E2/ERα-SMYD3-SHCBP1 oncogenic axis to maintain genome integrity, minimize the risk of malignant transformation and ensure normal cell proliferation. Our study uncovered a mechanism underlying the interactions among BRCA1, E2/ERα, and downstream signaling in suppressing PABC and non-PABC tumor formation.

E2/ERα signaling has been implicated in PABC. However, the mechanism by which it acts is unclear, as most PABCs do not express ERα [6]. Thus, it is hypothesized that the oncogenic effect of high levels of E2/ERα might act on stromal tissue rather than on mammary epithelial cells directly [64]. We have previously shown that mammary epithelial cells and mammary tumors in Brca1 mutant mice at early stages are ER-positive and that the expression of ERα is gradually reduced during cancer progression [65]. Similarly, it was shown that 71% of BRCA1-deficient breast cancers at Grade 1 are ER-positive, and the positivity was reduced to approximately 16% at Grade 3 [66]. To directly test the potential impact of estrogen on Brca1 mutant cells and mice, we had previously treated *Brca1*^*Δ11Δ11*^*;p53*^*+/−*^ cells and mice with estrogen and found it enhanced cell proliferation and mammary branch morphogenesis [65]. Thus, we believe that BRCA1 and ERα should play important roles in PABC, at least, in its early developmental phases. In this regard, we have recently shown that E2/ERα signaling positively regulates DNA replication, while Brca1 antagonizes it by inhibiting the expression of replisome-related genes in both mouse and human mammary epithelial cells, and E2/ERα signaling and Brca1 jointly play a critical role for the rapid proliferation while maintaining the genome stability of mammary epithelial cells during pregnancy.

We identified SMYD3 as a mediator of BRCA1-ERα signaling. It has been shown that SMYD3 is recruited to the proximal promoter regions of ERα target genes and is responsible for the accumulation of H3K4me2 and H3K4me3 at the induced ERα target genes [67]. Because Smyd3 is highly expressed in many types of cancers and plays critical roles in cell cycle alteration, apoptosis, and EMT [43, 68–70], increased ERα-SMYD3 signaling before, during, and post pregnancy could significantly contribute to PABC and breast cancer formation in general. Of note, our analysis of cBioportal data revealed SMYD3 amplification in up to 24% of breast cancers. The amplification provides further support for the role in general oncogenic process of SMYD3, which may be independent of BRCA1 mutations, as such mutations are only identified in a small fraction (2–4%) of human breast cancers [2, 3]. Our data also revealed a positive correlation between the expression of Smyd3, Shcbp1, H3K4me3 and Kras-MAPK in the mammary glands of Brca1^MKO^ mice at all distinct developmental stages examined. These findings suggest that Smyd3 could be a general regulator of Shcbp1, leading to the dynamics of increase of H3K4me3 and Smyd3 activation in both BRCA–/− and parous mammary epithelial cells in premalignant stages and general tumor progression process.

In summary, we have studied the functions of BRCA1 and its downstream signaling in breast tumor formation in mice. Brca1 deficiency together with ERα signaling enhances SMYD3-SHCBP1 expression, leading to more profound mammary tumor formation in parous mice than virgin mice. We believe that pregnancy is stressful and prone to DNA damage due to fast cell proliferation. This is especially true in the absence or impaired function of Brca1, which serves as a guardian during pregnancy in maintaining genome integrity by antagonizing the E2/ERα-SMYD3-SHCBP1 oncogenic axis and prohibiting replication catastrophe. E2/ERα-SMYD3, SHCBP1, H3K4me3 and Kras-MAPK in all developmental stages of mammary tissues in the Brca1^MKO^ mouse and parous mammary epithelial cells and serve as a cancer driver in general, although a stronger effect is observed in early pregnancy because of the higher levels of E2/ERα signaling. Finally, our study also uncovered therapeutic options for suppressing tumor formation by suppressing Smyd3-Shcbp1 signaling using a combination of trametinib and αPD1 treatment.
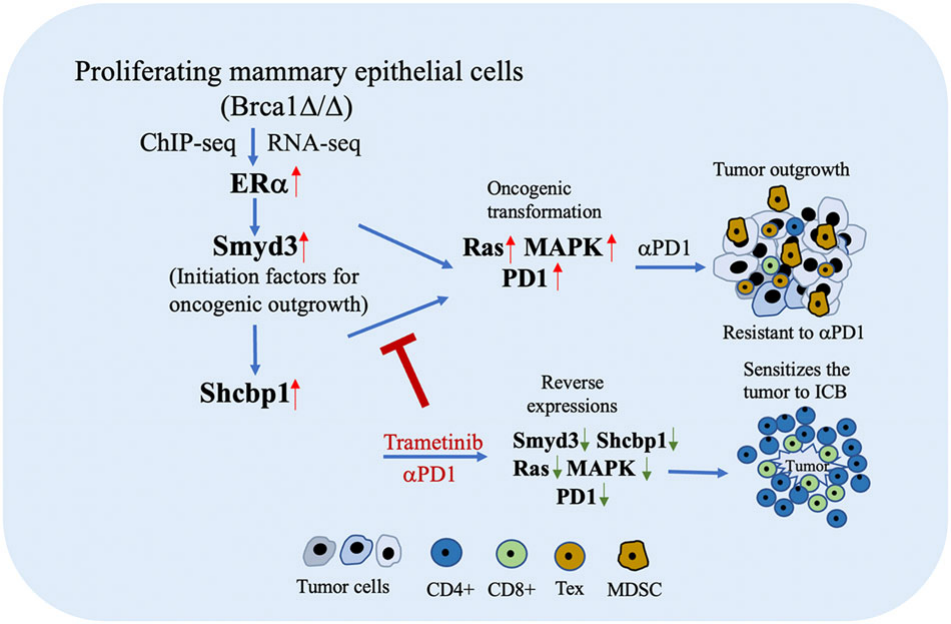


## Materials and methods

### Animal models

All experimental animals and protocols were approved by the Animal Facility of the University of Macau. Survival curves and tumor growth were observed in whole-body exon 11 deletion Brca1 knockout female mice rescued by *p53*^*+/−*^ mutation (*Brca1*^*Δ11/11Δ*^*;p53*^*+/−*^) and Brca1 double heterozygous female mice (*Brca1*^*Δ11/+*^*;p53*^*+/−*^) that underwent two pregnancies or were at the virgin stage. Brca1-Flag mice were generated by insertion of the 72 bp 3X-falg sequence immediately before the stop codon of WT ES and Brca1-mutant ES cells by TALEN technology. Two ES clones went through germline and the Flag/Flag mice were developmentally normal up until 18 months of age. The Flag mice used in this study were 3 months old. Wild type and Brca1 mutant mouse mammary glands used in chromatin immunoprecipitation (ChIP) assays were isolated from the *3XFlag-Brca1*^*+/+*^ and Brca1 conditional knockout mice (*Brca1*^*co/co*^*;p53*^*+/−*^*;MMTV-Cre* mice) at both virgin and 12.5 (P12) days of pregnancy. For the xenograft studies, 6-week-old female nude mice were implanted with B477 cells and G600 (2 × 10^5^ cells), MDA-MB-231 (2 × 10^6^ cells) or HCC1937 (5 × 10^6^ cells) cells through a mammary fat pad (MFP) injection. LMBG (545) (5 × 10^6^ cells) and HP5712 (1 × 10^6^ cells) cells were injected into FVB female mice via MFP injection. For the pregnancy mouse xenograft model, 6-week-old female nude mice were subjected to MFP injection after fertilization. For the drug treatment mouse model, PD1 antibody was diluted with PBS, and then mice were administered 0.1 mg per mouse 3 times via intraperitoneal injection. Trametinib (Sellectchem, USA, Cat No. S2673) was dissolved in 4% DMSO and corn oil and then mixed with 0.5% hydroxypropyl methylcellulose (HPMC, Sigma, USA) and 0.2% Tween-80. Mice were administered 0.5 mg/kg every other day by oral gavage. For the T-cell depletion mouse model, anti-mouse CD8a antibody was diluted with PBS and treated with 0.3 mg per mouse 3 times via intraperitoneal injection.

### Human breast cancer samples

The human breast cancers were originated from Armed Forces Institute of Pathology and American Registry of Pathology (AFIP & ARP) [71, 72]. All PABC were collected during pregnancy or within one year of delivery and were received from multiple international contributors with IRB approvals. Consecutive sections at 4-5 μm thickness were prepared from formalin-fixed, paraffin-embedded tissue blocks from 14 patients with PABC, and 14 cases of stage, grade, and age matched non-PABC were prepared and placed on positively charged slides.

### Cell culture and viral infection

The G600 cell line was derived from luminal mammary epithelial cells of a Brca1^Δ11/Δ11^; p53^+/−^ female mouse at premalignant stage. The B477 cell line was derived from luminal mammary epithelial cells of a age matched Brca1^+/+^; p53^+/−^ mouse. These cells were immortalized and malignantly transformed after continuing culture and spontaneously deleted their remaining WT copy of p53 gene [33, 73]. The 545 cells were derived from Brca1^MKO^ tumor mice with FVB background. The HP5712 cell line was derived from a spontaneous mammary tumor in a 22- months old FVB parous mouse [58], and it also expresses ERα at a moderate level in both cell culture and allograft tumors. HEK293T, MDA-MB-231, and HCC1937 cell lines were acquired from ATCC. All cell lines were grown in DMEM (Life Technologies, Carlsbad, CA) supplemented with 10% (v/v) fetal bovine serum (FBS) (Sigma, St. Louis, MO), 1% L-glutamine (Life Technologies), 0.6% Pen-Strep (Life Technologies), and 10 μg/ml insulin (Invitrogen, Carlsbad, CA). For lentiviral infections, specific sgRNA sequences (listed in Table 1) were individually cloned and inserted into the V2 vector (Addgene plasmid No. 52961) by using a general protocol. 293 T cells were seeded on 10-cm dishes and transfected with psPAX2 (3 μg), pMD2.G (9 μg) and lentiCRISPRV2 plasmid (12 μg). The lentivirus-containing supernatants were collected 48 hours after transfection and filtered through a 0.45-µm strainer (Corning, NY). Then, the transfected cells were selected with puromycin after 72 hours.

**Table 1:** Primers used for ChIP-qPCR, qRT-PCR, and PCR.

Brca1 ChIP F	ACTTCAAAGGGGGACTTGGT
Brca1 ChIP R	TGAGCCACAAGACTCCAATG
Esr1 ChIP F	TAGATCAGCCTGGGTTAGGG
Esr1 ChIP R	TGCCTGGAGGCTGTCTCTAT
Shcbp1 ChIP F	CATCCAAGTTTGCCTTGCTGAT
Shcbp1 ChIP R	GCGTCAGTACTCGACTAGAGG
q-Smyd3 F	GCGTTGTTCTCAATGCCGAA
q-Smyd3 R	GGGCTTTTCATCCATCAGCTT
q-Psmd9 F	CAGACTGATTCCAACCCGCT
q-Psmd9 R	AGAGAACGAGGAAACGCCTC
q-Esr1 F	ATTATGGGGTCTGGTCCTGC
q-Esr1 R	TCTTTCCGTATGCCGCCTTT
q-Pgr F	TATGGCGTGCTTACCTGTGG
q-Pgr R	ACTTACGACCTCCAAGGACCA
q-Ncoa1 F	CCAGTCCTTCGGCAGATGAG
q-Ncoa1 R	AGAAGTCCTGAGTCCAGAGGC
q-Crebbp F	CTGTCCTGTTTGCCTCCCTTT
q-Crebbp R	AAGTGGCATTCTGTTGCCCT
q-Arid1b F	CTCCCCTGCGAGTATTCCAG
q-Arid1b R	TGCCTGTCATAAAACCTCTTTCC
q-Hist1h2be F	GTACCCGCAGTACCACCTTT
q-Hist1h2be R	AGTGTGAAACGTTCGGGGTT
q-Pik3ca F	AAAATGGCGACGACTTACGG
q-Pik3ca R	CGATGAGACCCACACAGTCC
q-Chd1l F	AGACCTGCCAGACTATCGCT
q-Chd1l R	TCCAGTTGCTCAAAACAGACAA
q-Hmgn2 F	CGCTGTATCCCCGTGCTCA
q-Hmgn2 R	AGGGGCCTTTTTAGGTTTGGG
q-Asf1b F	GGTCCTGCTTCATGCATTCT
q-Asf1b R	TAAGGGCTTAGGATAGAGGTGG
q-Pcsk6 F	GTACACAGCTCCTTCCATCCAA
q-Pcsk6 R	GGTCTGCATTGGGACCATCA
q-Mapk8 F	GTGGAATCAAGCACCTTCACT
q-Mapk8 R	TCCTCGCCAGTCCAAAATCAA
q-Shcbp1 F	CTCCATTGAGTTGGAAGGATATGG
q-Shcbp1 R	TGTGGTCTTGCCATGGTGAAT
q-Kras F	GCCTATGGTCCTGGTAGGGA
q-Kras R	ATCGTCAACACCCTGTCTTGT
q-Src F	CAATGCCAAGGGCCTAAATGT
q-Src R	TGTTTGGAGTAGTAAGCCACGA
q-Sos1 F	TGTTCGATTCTGACCACTCGG
q-Sos1 R	TTAGACATAATCTTGGATGGGGAGG
q-Grb2 F	GGAAAGATGGCTTCATCCCCA
q-Grb2 R	TTCCAAACTTGACGGACAGGGA
q-PD1 F	AGAGCTCGTGGTAACAGAGAGA
q-PD1 R	CAGGGATACCCACTAGGGCA
q-Pd-L1 F	ACTTGCTACGGGCGTTTACT
q-Pd-L1 R	GTCCAGCTCCCGTTCTACAG
q-Pik3cb F	GGCAGCAAGAGACTGGGAG
q-Pik3cb R	ACAGCCCCCATTTCAGTCTT
q-N-Myc F	AAGTCACCTTGTTCCGGTCC
q-N-Myc R	TTCCCAGGGGCATCAAATGG
q-CrkL F	TTTGCCGGCCTTGTTAGAGT
q-CrkL R	TGTGTGAAGGGGAAAAGCCC
q-Wnt10b F	GGTGGCTGTAACCACGACAT
q-Wnt10b R	GCCTGACGTTCCATGGCATT
q-Riz F	CGCTCTGGGCTCATGTAATCA
q-Riz R	AATCCGAGTCTTGTCGACCG
q-Ccng1 F	GCCCATGATAATGGCCTCAGA
q-Ccng1 R	CTCAGTCCAACACACCCAAGA
q-Cdk2 F	AATCAAGGGCCCGTTTGGAG
q-Cdk2 R	CAGACCAGAGTGACGTGCAA
q-Map3k11 F	CTAGTACTCCCCCGGCACT
q-Map3k11 R	GCAATGCACGTTGGATCAGT
Mouse Smyd3-sg1-F	CACCGACTGCTCCATCGTATTCAAC
Mouse Smyd3-sg1-R	AAACGTTGAATACGATGGAGCAGTC
Mouse Smyd3-sg2-F	CACCGCCCGCACTGCACGCAGTAAG
Mouse Smyd3-sg2-R	AAACCTTACTGCGTGCAGTGCGGGC
Mouse Smyd3-sg3-F	CACCGACGCAGCCCGTTTCCCCGGT
Mouse Smyd3-sg3-R	AAACACCGGGGAAACGGGCTGCGTC
Human SMYD3-sg1-F	CACCGATACTGTAGTGCTAAGTGTC
Human SMYD3-sg1-R	AAACGACACTTAGCACTACAGTATC
Human SMYD3-sg2-F	CACCGCCAGACTCCGTTCGACTTCT
Human SMYD3-sg2-R	AAACAGAAGTCGAACGGAGTCTGGC
Human SMYD3-sg3-F	CACCGTTGCATTCCCGCTTGTGGTC
Human SMYD3-sg3-R	AAACGACCACAAGCGGGAATGCAAC
Mouse Shcbp1-sg1-F	CACCGCATCCCTGAAGGCGGTCGTA
Mouse Shcbp1-sg1-R	AAACTACGACCGCCTTCAGGGATGC
Mouse Shcbp1-sg2-F	CACCGCAAGCCTTACGACCGCCTTC
Mouse Shcbp1-sg2-R	AAACGAAGGCGGTCGTAAGGCTTGC
Mouse Shcbp1-sg3-F	CACCGCTCGGTGGAACCATAGCTAT
Mouse Shcbp1-sg3-R	AAACATAGCTATGGTTCCACCGAGC
Human SHCBP1-sg1-F	CACCGTGTAGCTACCGTGATAAACC
Human SHCBP1-sg1-R	AAAC GGTTTATCACGGTAGCTACAC
Human SHCBP1-sg2-F	CACCGCTGGCACACTAATGTGTTCA
Human SHCBP1-sg2-R	AAAC TGAACACATTAGTGTGCCAGC
Human SHCBP1-sg3-F	CACCGATGGCTGACGGGTCGCTGAC
Human SHCBP1-sg3-R	AAAC GTCAGCGACCCGTCAGCCATC
Brca1 C1 primer	CGTCTTGTGATGTGGGACTG
Brca1 C2 primer	CCTCACATAGGTAGAAGCTG

### Bulk RNA sequencing and differential gene expression

RNA was extracted as three biological triplicates from WT virgin (WTV) mice, Brca1^MKO^ virgin mice (MTV), WT mice at pregnancy day 12 (WTP12), and Brca1^MKO^ mice at pregnancy day 12 (MTP12), and bulk RNA-Seq was performed. The raw RNA sequencing data were cleaned using Trim Galore software to remove low-quality bases, and reads smaller than 36 bp were dropped [http://www.bioinformatics.babraham.ac.uk/projects/trim_galore/]. The paired-end reads were then aligned to the mouse genome reference mm10 via hisat2 [74] with default parameters. The mapping tool featureCounts [75] was used to count the number of reads of each gene. Differentially expressed genes were called through a negative binomial generalized linear model fitted to the median of ratios normalized values, as recommended in the DESeq2 R package manual [76]. Differentially expressed genes were decided by cutoffs of Benjamini‒Hochberg with adjusted p < 0.05 and log2FoldChanges at least 0.5 in absolute value.

### Chromatin immunoprecipitation (ChIP) assay

Fresh mouse mammary glands from both virgin and P12 mice were cut into pieces and digested for approximately 3 hours at 37 °C. Then, ChIP assays were carried out by using ~2.0 × 10^7^ freshly isolated mouse mammary epithelial cells. The single cell suspension was crosslinked by 1% (v/v) formaldehyde solution for 5 minutes at room temperature and stopped by glycine buffer. Then, the chromatin was fragmented by sonication using a truChIP Ultra-Low Chromatin Shearing Kit (Covaris, USA, 520154). Antibodies against mouse Flag (target Brca1), ERα and the histone markers H3K4me3 and H3K27Ac were used to immunoprecipitate chromatin DNA for ChIP sequencing using a LowCell ChIP KitTM (Diagenode, Japan, Cat No. C01010072, C01010073). The DNA library was prepared with the TruSeq ChIP Sample Prep Kit (Illumina, USA, 15034288, 15034289). Mouse and rabbit IgG were used as negative controls, and the results were normalized to input. The antibodies used for ChIP are listed in Table 2. Then, the ChIP DNA library was mixed and sent out for sequencing. The quality of the raw data was checked using the FastQC program. Raw reads were aligned to the mouse reference genome GRCm38.84 using Bowtie2 software. Alignment output from Bowtie2 was converted to BAM format using the SAMtools software suite. For ChIP-seq data visualization, mapped reads were extended to 200 bp in the 5’- > 3’ direction using the ‘macs2 pileup’ command and scaled to 1 million mapped reads using the ‘macs2 bdgopt’ command from the MACS2 tool. MACS2 tools were used for peak calling with default parameters, and the q-value (minimum false discovery rate) cutoff for peak detection was set to 0.05. UCSC Kent utils’ programs ‘bedSort’ and ‘bedGraphToBigWig’ were used to convert the genome coverage files from BedGraph to BigWig format. These ChIP-seq genome coverage files were visualized using Integrative Genomics Viewer software.

**Table 2:** Antibodies used for ChIP, IHC, IF, WB and in vivo mouse study.

Item description	Source	Model/Cat. #	IHC/IF	WB
Monoclonal Anti-Flag M2 Antibody (ChIP)	Sigma	F1804-1MG		
ER-α (MC-20) Antibody (ChIP)	Santa Cruz	sc-542		1:500
H3K4me3 Antibody (ChIP)	Cell Signaling	#9751S		
H3K27Ac Antibody (ChIP)	EMD Millipore	07-360		
Normal Rabbit Antibody	Santa Cruz	sc-2027		
Normal Mouse Antibody	Santa Cruz	sc-2025		
H3K4me3 Antibody	Abcam	Ab12209	1:200	1:1000
SMYD3 Antibody	Gene Tex	GTX121945	1:200	1:1000
H3K9me3 Antibody	EMD Millipore	07-442	1:200	
BRCA1 Antibody (C-20)	Santa Cruz	sc-642	1:50	
BRCA1 Antibody (D-9)	Santa Cruz	sc-6954		1:100
BRCA1 Antibody	Santa Cruz	sc-135732		1:100
Ki67 Antibody	BioLegend	350502	1:50	
Anti-SHCBP1 Antibody	Abcam	ab184467		1:1000
KRAS Antibody	Abcam	ab216890		1:1000
p44/42 MAPK (Erk1/2) Antibody	Cell Signaling	#4695S		1:1000
Phospho-p44/42 MAPK (Erk1/2) Antibody	Cell Signaling	#4370L		1:1000
MEK1/2 (L38C12) Antibody	Cell Signaling	#4694S		1:1000
Phospho-MEK1/2 (Ser217/221) Antibody	Cell Signaling	#9154S		1:1000
GRB2 (C-7) Antibody	Santa Cruz	sc-8034		1:500
In Vivo MAb anti-mouse PD-1 (CD276)	BioX Cell	BE0146		
F4/80	Abcam	ab6640	1:200	
CD86	LSBio	LS-C392134	1:200	
CD206	BDPharmingn	AF2535	1:200	
PD1	Cell Signaling	84651S	1:200	
CD3	Santa Cruz	sc-20047	1:100	
In Vivo anti-mouse CD8a	BioX Cell	BP0117		
CD4	Invitrogen	14-9766-82	1:200	
CD8a	Cell Signaling	70306S	1:200	
PD1	Proteintech	66220-1-lg	1:200	

### Binding intensity analysis

The general binding intensity analysis in promoter regions was assayed by the software “deeptools” (https://deeptools.readthedocs.io/en/develop/index.html). Raw ChIP-seq data were converted into a bam file, and the “ngsplot” tool was used to quantify and test the intensity of H3K4me3.

### ChIP‒qPCR, qRT‒PCR, and PCR analysis

For ChIP‒qPCR, the immunoprecipitated chromatin DNA of mouse mammary gland tissues were assessed by qPCR using primers for Brca1 or ERα to amplify DNA regions. Immunoprecipitated chromatin DNA from B477 and G600 cells was generated by Shcbp1 primers. The ChIP primers used are listed in Table 1. For qRT‒PCR analysis, total RNA was isolated from tumors, fresh mouse mammary glands or cell lines with TRIzol reagent (Thermo Fisher Scientific, USA), and reverse transcription was carried out by using a QuantiTect reverse transcription kit (Qiagen, Germany, 205313). RT‒PCR was conducted with FastStart Universal SYBR Green Master Mix (Roche, Switzerland, 24759100) in a QuantStudio™ 7 Flex Real-Time PCR System (Thermo Fisher Scientific, USA). Relative quantification was assessed by normalization to 18S. The qRT‒PCR primers used are listed in Table 1.

### Protein extraction and Western blotting (WB) analysis

Tumors, mammary gland tissues and cells were washed with PBS and then lysed with RIPA buffer (50 mM Tris-HCl (pH 7.4), 1% NP-40, 150 mM NaCl, 0.1% SDS, 1% deoxycholic acid, 1 mM EDTA and 10% glycerol) supplemented with a protease inhibitor cocktail table (Roche, Switzerland, 04693159001) and phosphatase inhibitor cocktail table (Roche, Switzerland, 04906837001). Western blotting was performed by an ODYSSEY CLx system (LI-COR) with the corresponding antibodies (listed in Table 2). The band intensities were quantified with ImageJ (National Institutes of Health (NIH), MD, USA). Relative quantification was acquired by normalization to β-actin.

### H&E, immunohistochemistry (IHC) and immunofluorescence (IF) staining

Tumors or mammary gland tissues were fixed with 4% v/v formaldehyde solution and embedded in paraffin. Then, the paraffin sections were deparaffinized and rehydrated with xylene and gradient ethyl alcohol. The deparaffinized sections were cooked with 10 ml 10× R-Buffer-A (Electron Microscopy Sciences, Cat No. 62706-10) in 90 ml dH_2_O followed by staining with the corresponding antibodies according to a general method. Images were obtained with an Olympus IX83 inverted microscope (Olympus Corp., Japan) or a Nikon A1R confocal system (Nikon Corp., Japan). Fluorescent signals and DAB intensities were analyzed with ImageJ (NIH, MD, USA). The antibodies used in IF and IHC staining are listed in Table 2.

### Transfection and luciferase reporter assay

Cells were seeded in 24-well plates one day before transfection, and 0.8 µg of plasmid was transfected with Lipofectamine ^TM^ 3000 (Thermo Fisher Scientific, USA, L3000015) based on the manufacturer’s instructions. The cells were cultured for 24 hours posttransfection and then treated with 100 nM 17β-estradiol (Sigma, USA) for 24 hours. The luciferase activity was assessed by a dual luciferase reporter assay system (Promega, USA, E1960). The results were normalized to Renilla luciferase activity.

### Cell proliferation assay

Cells (3000 per well) were seeded on 96-well plates. Then, they were cultured and observed for one week with an IncuCyte® Live Cell Analysis System. Cell proliferation curves were then generated.

### Tumor slice culture

Fresh tumor tissues were trimmed to 250 µm of appropriate size and then seeded on reconstituted collagen gel consisting of three solutions. Solution A: cultures 3D culture matrix type 1 collagen (R&D Systems, Minneapolis, USA). Solution B: concentrated sterile culture medium (10×) composed of Ham’s F-12 nutrient mix powder (Gibco™, USA) dissolved in Milli-Q water and filtered by a 0.22-µm strainer (Corning, NY). Solution C: sterile reconstitution buffer (0.05 M NaOH, 200 mM HEPES and 2.2 g NaHCO3 dissolved in 100 mL sterile Milli-Q water). All solutions were stored at 4 °C. To prepare the reconstituted collagen gel, Solutions A, B, and C were mixed at a ratio of 8:1:1 (v/v/v). Then, the seeded tumor slices were cultured with F-medium, which consisted of Dulbecco’s modified Eagle’s medium (DMEM), Ham’s F-12 nutrient mix powder, 5 μg/mL insulin, 10 μg/mL gentamicin, 250 ng/mL amphotericin B, 0.1 nM cholera toxin, 25 ng/mL hydrocortisone, 0.125 ng/mL EGF, and 10 μM ROCK inhibitor. The SMYD3 inhibitor BCI-121 (Chemicals, Shanghai, China, DC10885) was dissolved in DMSO and stored at −80 °C before use. BCI-121 was then diluted to 50 μΜ, 100 µM, 200 µM, and 300 µM with the culture medium.

### T-cell suppression assay

T cells were isolated from spleen tissues of 2-month-old normal FVB mice by the CD8a + T-Cell Isolation Kit (Miltenyi Biotec, 130-104-075). MDSCs were isolated by using the Myeloid-Derived Suppressor Cell Isolation Kit (Miltenyi Biotec, 130-094-538) from spleen tissues of FVB mice implanted with HP5712-Ctr, HP5712-OE-Smyd3, HP5712-sgSmyd3 or HP5712-OE-Smyd3/sgShcbp1 cells. The collected T cells were stained with 10 µM CFSE (Invitrogen, C34570) for 10 min and then quenched with complete cell culture medium on ice for 5 min. MDSCs were cocultured with T cells at a ratio of 0:1 or 1:1 in CD3/CD28-coated 48-well plates. Cells were incubated at 37 °C for 72 h and then subjected to FACS analysis.

### Mass cytometry (CyTOF)

Mouse mammary gland and mammary tumor tissues were cut into small pieces and digested for approximately one hour at 37 °C. Single cells were then collected by passing through a 40 μm cell strainer and resuspended in prewarmed serum-free medium after HBSS washing. Single cells (1 × 10^7^) were mixed with 10 μM cisplatin working solution and incubated at RT for 5 min. Following washing with Maxpar Cell Staining Buffer, 1 ~ 3 × 10^6^ cells were then incubated with an antibody cocktail (CD45-147Sm: DVS-Fluidigm, Catalog 3147003B; CD3e-152Sm: DVS-Fluidigm, Catalog 3152004B; CD4-145Nd: DVS-Fluidigm, Catalog 3145002B; CD8a-146Nd: DVS-Fluidigm, Catalog 3146003B; Ly6G-141Pr: DVS-Fluidigm, Catalog 3141008B; Ly6C-162Dy: DVS-Fluidigm, Catalog 3162014B; CD28-151Eu: DVS-Fluidigm, Catalog 3151005B; CD11b-148Nd: DVS-Fluidigm, Catalog 3148003B) according to a commercialized method. Then, the antibody-stained cells were mixed with Cell-ID Intercalator-Ir solution diluted with Maxpar Fix and Perm Buffer and incubated at 4 °C overnight. After washing with Maxpar Cell Staining Buffer and Maxpar water, 2.5 ~ 5 × 10^5^/ml cells were adjusted by EQ^TM^ Four Element Calibration buffer (Cat. #201078), and the data were acquired on a CyTOF2 System Mass Cytometer (FluidigmHelios). Then, the different cell populations were analyzed by FlowJo 10.4 and calculated by GraphPad Prism 9 software. The CyTOF data were analyzed with the R-based pipeline cytofWorkflow.

### Statistical analysis

Statistical analysis was carried out using the Welch t test or nonparametric test with normality and the Shapiro‒Wilk lognormality test. A p value < 0.05 was considered to indicate statistical significance, represented as *p* < 0.05*, *p* < 0.01**, *p* < 0.001***, and *p* < 0.0001****. Values are expressed as the mean (SD) or mean ± SEM. The sample size (n) was provided in the corresponding content for individual statistical analysis. Each dataset was analyzed ≥3 times to ensure repeatability.

## Supplementary information

Supplementary Figures

Legends for Supplementary data 1-8

Supplementary data 1

Supplementary data 2

Supplementary data 3

Supplementary data 4

Supplementary data 5

Supplementary data 6

Supplementary data 7

Supplementary data 8

Full length western blots

## Data Availability

ChIP-seq data and bulk RNA-seq data generated in this study have been deposited in the National Institute Center for Biotechnology Information Sequence Read Archive database under accession numbers PRJNA990527 for the RNA sequence and PRJNA799920 for the ChIP sequence, respectively.

## References

[1] Ferlay J, Colombet M, Soerjomataram I, Parkin DM, Pineros M, Znaor A, et al. Cancer statistics for the year 2020: an overview. Int J Cancer. 2021;149:778–89. 10.1002/ijc.33588 33818764

[2] Hemel D, Domchek SM. Breast cancer predisposition syndromes. Hematol Oncol Clin North Am. 2010;24:799–814. 20816575 10.1016/j.hoc.2010.06.004

[3] Hall MJ, Reid JE, Burbidge LA, Pruss D, Deffenbaugh AM, Frye C, et al. BRCA1 and BRCA2 mutations in women of different ethnicities undergoing testing for hereditary breast-ovarian cancer. Cancer. 2009;115:2222–33. 19241424 10.1002/cncr.24200 PMC2771545

[4] Azim HA Jr, Santoro L, Russell-Edu W, Pentheroudakis G, Pavlidis N, Peccatori FA. Prognosis of pregnancy-associated breast cancer: a meta-analysis of 30 studies. Cancer Treat Rev. 2012;38:834–42. 22785217 10.1016/j.ctrv.2012.06.004

[5] Kakoulidis I, Skagias L, Politi E. Pregnancy associated breast cancer (PABC): aspects in diagnosis. Breast Dis. 2015;35:157–66. 26406540 10.3233/BD-150408

[6] Paris I, Di Giorgio D, Carbognin L, Corrado G, Garganese G, Franceschini G, et al. Pregnancy-associated breast cancer: a multidisciplinary approach. Clin Breast Cancer. 2021;21:e120–7. 32778512 10.1016/j.clbc.2020.07.007

[7] Mathelin C, Annane K, Treisser A, Chenard MP, Tomasetto C, Bellocq JP, et al. Pregnancy and post-partum breast cancer: a prospective study. Anticancer Res. 2008;28:2447–52. 18751433

[8] Rodriguez AO, Chew H, Cress R, Xing G, McElvy S, Danielsen B, et al. Evidence of poorer survival in pregnancy-associated breast cancer. Obstet Gynecol. 2008;112:71–8. 18591310 10.1097/AOG.0b013e31817c4ebc

[9] Polyak K. Pregnancy and breast cancer: the other side of the coin. Cancer Cell. 2006;9:151–3. 16530699 10.1016/j.ccr.2006.02.026

[10] Johansson AL, Andersson TM, Hsieh CC, Cnattingius S, Lambe M. Increased mortality in women with breast cancer detected during pregnancy and different periods postpartum. Cancer epidemiology, biomarkers & prevention: a publication of the American Association for Cancer Research, cosponsored by the American Society of Preventive. Oncology. 2011;20:1865–72. 10.1158/1055-9965.EPI-11-0515 21750168

[11] Jernstrom H, Lerman C, Ghadirian P, Lynch HT, Weber B, Garber J, et al. Pregnancy and risk of early breast cancer in carriers of BRCA1 and BRCA2. Lancet. 1999;354:1846–50. 10584720 10.1016/s0140-6736(99)04336-6

[12] Johannsson O, Loman N, Borg A, Olsson H. Pregnancy-associated breast cancer in BRCA1 and BRCA2 germline mutation carriers. Lancet. 1998;352:1359–60. 9802282 10.1016/S0140-6736(05)60750-7

[13] Hou N, Ogundiran T, Ojengbede O, Morhason-Bello I, Zheng Y, Fackenthal J, et al. Risk factors for pregnancy-associated breast cancer: a report from the Nigerian Breast Cancer Study. Ann Epidemiol. 2013;23:551–7. 23880155 10.1016/j.annepidem.2013.06.008 PMC3770152

[14] Ruiz R, Herrero C, Strasser-Weippl K, Touya D, St Louis J, Bukowski A, et al. Epidemiology and pathophysiology of pregnancy-associated breast cancer: a review. Breast. 2017;35:136–41. 28732325 10.1016/j.breast.2017.07.008

[15] Andrieu N, Goldgar DE, Easton DF, Rookus M, Brohet R, Antoniou AC, et al. Pregnancies, breast-feeding, and breast cancer risk in the International BRCA1/2 Carrier Cohort Study (IBCCS). J Natl Cancer Inst. 2006;98:535–44. 16622123 10.1093/jnci/djj132 PMC2094011

[16] Valentini A, Lubinski J, Byrski T, Ghadirian P, Moller P, Lynch HT, et al. The impact of pregnancy on breast cancer survival in women who carry a BRCA1 or BRCA2 mutation. Breast Cancer Res Treat. 2013;142:177–85. 24136669 10.1007/s10549-013-2729-1 PMC3940343

[17] Cullinane CA, Lubinski J, Neuhausen SL, Ghadirian P, Lynch HT, Isaacs C, et al. Effect of pregnancy as a risk factor for breast cancer in BRCA1/BRCA2 mutation carriers. Int J Cancer. 2005;117:988–91. 15986445 10.1002/ijc.21273

[18] Tu Z, Aird KM, Zhang R. Chromatin remodeling, BRCA1, SAHF and cellular senescence. Cell Cycle. 2013;12:1653–4. 23673322 10.4161/cc.24986 PMC3713120

[19] Wu W, Koike A, Takeshita T, Ohta T. The ubiquitin E3 ligase activity of BRCA1 and its biological functions. Cell Div. 2008;3:1. 18179693 10.1186/1747-1028-3-1 PMC2254412

[20] Deng CX. BRCA1: cell cycle checkpoint, genetic instability, DNA damage response, and cancer evolution. Nucleic Acids Res. 2006;34:1416–26. 16522651 10.1093/nar/gkl010 PMC1390683

[21] Dine J, Deng CX. Mouse models of BRCA1 and their application to breast cancer research. Cancer Metastasis Rev. 2013;32:25–37. 23093327 10.1007/s10555-012-9403-7

[22] Fu X, Tan W, Song Q, Pei H, Li J. BRCA1 and breast cancer: molecular mechanisms and therapeutic strategies. Front Cell Dev Biol. 2022;10:813457. 35300412 10.3389/fcell.2022.813457 PMC8921524

[23] Li J, Shu X, Xu J, Su SM, Chan UI, Mo L, et al. S100A9-CXCL12 activation in BRCA1-mutant breast cancer promotes an immunosuppressive microenvironment associated with resistance to immunotherapy. Nat Commun. 2022;13:1481. 35304461 10.1038/s41467-022-29151-5 PMC8933470

[24] Lim E, Vaillant F, Wu D, Forrest NC, Pal B, Hart AH, et al. Aberrant luminal progenitors as the candidate target population for basal tumor development in BRCA1 mutation carriers. Nat Med. 2009;15:907–13. 19648928 10.1038/nm.2000

[25] Molyneux G, Geyer FC, Magnay FA, McCarthy A, Kendrick H, Natrajan R, et al. BRCA1 basal-like breast cancers originate from luminal epithelial progenitors and not from basal stem cells. Cell Stem Cell. 2010;7:403–17. 20804975 10.1016/j.stem.2010.07.010

[26] Ng T, Irshad S, Stebbing J. BRCA1 mutations and luminal-basal transformation. Oncogene. 2013;32:2712–4. 22926516 10.1038/onc.2012.379

[27] Kauff ND, Satagopan JM, Robson ME, Scheuer L, Hensley M, Hudis CA, et al. Risk-reducing salpingo-oophorectomy in women with a BRCA1 or BRCA2 mutation. N Engl J Med. 2002;346:1609–15. 12023992 10.1056/NEJMoa020119

[28] Bachelier R, Li C, Qiao W, Furth PA, Lubet RA, Deng CX. Effects of bilateral oophorectomy on mammary tumor formation in breast cancer associated gene 1 (Brca1) mutant mice. Oncol Rep. 2005;14:1117–20. 16211273

[29] Prall OW, Rogan EM, Sutherland RL. Estrogen regulation of cell cycle progression in breast cancer cells. J Steroid Biochem Mol Biol. 1998;65:169–74. 9699870 10.1016/s0960-0760(98)00021-1

[30] Calderon-Margalit R, Paltiel O. Prevention of breast cancer in women who carry BRCA1 or BRCA2 mutations: a critical review of the literature. Int J Cancer. 2004;112:357–64. 15382059 10.1002/ijc.20429

[31] Gadducci A, Biglia N, Sismondi P, Genazzani AR. Breast cancer and sex steroids: critical review of epidemiological, experimental and clinical investigations on etiopathogenesis, chemoprevention and endocrine treatment of breast cancer. Gynecol Endocrinol. 2005;20:343–60. 16019385 10.1080/09513590500128492

[32] Xu X, Wagner KU, Larson D, Weaver Z, Li C, Ried T, et al. Conditional mutation of Brca1 in mammary epithelial cells results in blunted ductal morphogenesis and tumour formation. Nat Genet. 1999;22:37–43. 10319859 10.1038/8743

[33] Xu X, Chen E, Mo L, Zhang L, Shao F, Miao K, et al. BRCA1 represses DNA replication initiation through antagonizing estrogen signaling and maintains genome stability in parallel with WEE1-MCM2 signaling during pregnancy. Hum Mol Genet. 2019;28:842–57. 30445628 10.1093/hmg/ddy398 PMC6381318

[34] Xu X, Weaver Z, Linke SP, Li C, Gotay J, Wang XW, et al. Centrosome amplification and a defective G2-M cell cycle checkpoint induce genetic instability in BRCA1 exon 11 isoform-deficient cells. Mol Cell. 1999;3:389–95. 10198641 10.1016/s1097-2765(00)80466-9

[35] Xu X, Qiao W, Linke SP, Cao L, Li WM, Furth PA, et al. Genetic interactions between tumor suppressors Brca1 and p53 in apoptosis, cell cycle and tumorigenesis. Nat Genet. 2001;28:266–71. 11431698 10.1038/90108

[36] Miao K, Lei JH, Valecha MV, Zhang A, Xu J, Wang L, et al. NOTCH1 activation compensates BRCA1 deficiency and promotes triple-negative breast cancer formation. Nat Commun. 2020;11:3256. 32591500 10.1038/s41467-020-16936-9 PMC7320176

[37] Cao L, Kim S, Xiao C, Wang RH, Coumoul X, Wang X, et al. ATM-Chk2-p53 activation prevents tumorigenesis at an expense of organ homeostasis upon Brca1 deficiency. EMBO J. 2006;25:2167–77. 16675955 10.1038/sj.emboj.7601115 PMC1462967

[38] Cao L, Xu X, Bunting SF, Liu J, Wang RH, Cao LL, et al. A selective requirement for 53BP1 in the biological response to genomic instability induced by Brca1 deficiency. Mol Cell. 2009;35:534–41. 19716796 10.1016/j.molcel.2009.06.037 PMC3392030

[39] Wu X, Xu Q, Chen P, Yu C, Ye L, Huang C, et al. Effect of SMYD3 on biological behavior and H3K4 methylation in bladder cancer. Cancer Manag Res. 2019;11:8125–33. 31564972 10.2147/CMAR.S213885 PMC6730607

[40] Walker VR, Korach KS. Estrogen receptor knockout mice as a model for endocrine research. ILAR J. 2004;45:455–61. 15454684 10.1093/ilar.45.4.455

[41] Yip CH, Rhodes A. Estrogen and progesterone receptors in breast cancer. Future Oncol. 2014;10:2293–301. 25471040 10.2217/fon.14.110

[42] Hamamoto R, Furukawa Y, Morita M, Iimura Y, Silva FP, Li M, et al. SMYD3 encodes a histone methyltransferase involved in the proliferation of cancer cells. Nat Cell Biol. 2004;6:731–40. 15235609 10.1038/ncb1151

[43] Bernard BJ, Nigam N, Burkitt K, Saloura V. SMYD3: a regulator of epigenetic and signaling pathways in cancer. Clin Epigenetics. 2021;13:45. 33637115 10.1186/s13148-021-01021-9 PMC7912509

[44] Giakountis A, Moulos P, Sarris ME, Hatzis P, Talianidis I. Smyd3-associated regulatory pathways in cancer. Semin Cancer Biol. 2017;42:70–80. 27554136 10.1016/j.semcancer.2016.08.008

[45] Xu N, Wu YP, Yin HB, Chen SH, Li XD, Xue XY, et al. SHCBP1 promotes tumor cell proliferation, migration, and invasion, and is associated with poor prostate cancer prognosis. J Cancer Res Clin Oncol. 2020;146:1953–69. 32447485 10.1007/s00432-020-03247-1 PMC11804494

[46] Wang J, Yang Z, Zhang C, Ouyang J, Zhang G, Wu C. A four-gene signature in the tumor microenvironment that significantly associates with the prognosis of patients with breast cancer. Gene. 2020;761:145049. 32791092 10.1016/j.gene.2020.145049

[47] Oshi M, Katsuta E, Yan L, Ebos JML, Rashid OM, Matsuyama R, et al. A novel 4-gene score to predict survival, distant metastasis and response to neoadjuvant therapy in breast cancer. Cancers. 2020;12:1148. 10.3390/cancers12051148 PMC7281399 32370309

[48] Zhou Q, Liu X, Lv M, Sun E, Lu X, Lu C. Genes that predict poor prognosis in breast cancer via bioinformatical analysis. BioMed Res Int. 2021;2021:6649660. 33959662 10.1155/2021/6649660 PMC8075678

[49] Liu N, Zhang GD, Bai P, Su L, Tian H, He M. Eight hub genes as potential biomarkers for breast cancer diagnosis and prognosis: a TCGA-based study. World J Clin Oncol. 2022;13:675–88. 36160462 10.5306/wjco.v13.i8.675 PMC9476610

[50] Zhao X, Lin J. Construction and validation of a prognostic model based on mRNAsi-related genes in breast cancer. Comput Math Methods Med. 2022;2022:6532591. 36267313 10.1155/2022/6532591 PMC9578885

[51] Wu M, Lu L, Dai T, Li A, Yu Y, Li Y, et al. Construction of a lncRNA-mediated ceRNA network and a genomic-clinicopathologic nomogram to predict survival for breast cancer patients. Cancer Biomark. 2023;36:83–96. 36591654 10.3233/CBM-210545 PMC12412795

[52] Zhang GY, Ma ZJ, Wang L, Sun RF, Jiang XY, Yang XJ, et al. The role of Shcbp1 in signaling and disease. Curr Cancer Drug targets. 2019;19:854–62. 31250756 10.2174/1568009619666190620114928

[53] Song RX, McPherson RA, Adam L, Bao Y, Shupnik M, Kumar R, et al. Linkage of rapid estrogen action to MAPK activation by ERalpha-Shc association and Shc pathway activation. Mol Endocrinol. 2002;16:116–27. 11773443 10.1210/mend.16.1.0748

[54] Rubio-Tomas T. Novel insights into SMYD2 and SMYD3 inhibitors: from potential anti-tumoural therapy to a variety of new applications. Mol Biol Rep. 2021;48:7499–508. 34510321 10.1007/s11033-021-06701-6

[55] Xing F, Liu YC, Huang S, Lyu X, Su SM, Chan UI, et al. Accelerating precision anti-cancer therapy by time-lapse and label-free 3D tumor slice culture platform. Theranostics. 2021;11:9415–30. 34646378 10.7150/thno.59533 PMC8490519

[56] Beheshti A, Wage J, McDonald JT, Lamont C, Peluso M, Hahnfeldt P, et al. Tumor-host signaling interaction reveals a systemic, age-dependent splenic immune influence on tumor development. Oncotarget. 2015;6:35419–32. 26497558 10.18632/oncotarget.6214 PMC4742115

[57] Quail DF, Joyce JA. Microenvironmental regulation of tumor progression and metastasis. Nat Med. 2013;19:1423–37. 24202395 10.1038/nm.3394 PMC3954707

[58] Zhou JB, Tang D, He L, Lin S, Lei JH, Sun H, et al. Machine learning model for anti-cancer drug combinations: analysis, prediction, and validation. Pharmacol Res. 2023;194:106830. 37343647 10.1016/j.phrs.2023.106830

[59] Mazur PK, Reynoird N, Khatri P, Jansen PW, Wilkinson AW, Liu S, et al. SMYD3 links lysine methylation of MAP3K2 to Ras-driven cancer. Nature. 2014;510:283–7. 24847881 10.1038/nature13320 PMC4122675

[60] Liu Y, Wang X, Liu Y, Yang J, Mao W, Feng C, et al. N4-acetylcytidine-dependent GLMP mRNA stabilization by NAT10 promotes head and neck squamous cell carcinoma metastasis and remodels tumor microenvironment through MAPK/ERK signaling pathway. Cell Death Dis. 2023;14:712. 37914704 10.1038/s41419-023-06245-6 PMC10620198

[61] Ai L, Xu A, Xu J. Roles of PD-1/PD-L1 pathway: signaling, cancer, and beyond. Adv Exp Med Biol. 2020;1248:33–59. 32185706 10.1007/978-981-15-3266-5_3

[62] Han Y. Liu D, Li L. PD-1/PD-L1 pathway: current researches in cancer. Am J Cancer Res. 2020;10:727–42. 32266087 PMC7136921

[63] Zhang X, Zhang R, Chen H, Wang L, Ren C, Pataer A, et al. KRT-232 and navitoclax enhance trametinib’s anti-Cancer activity in non-small cell lung cancer patient-derived xenografts with KRAS mutations. Am J Cancer Res. 2020;10:4464–75. 33415011 PMC7783771

[64] Gupta PB, Proia D, Cingoz O, Weremowicz J, Naber SP, Weinberg RA, et al. Systemic stromal effects of estrogen promote the growth of estrogen receptor-negative cancers. Cancer Res. 2007;67:2062–71. 17332335 10.1158/0008-5472.CAN-06-3895

[65] Li W, Xiao C, Vonderhaar BK, Deng CX. A role of estrogen/ERalpha signaling in BRCA1-associated tissue-specific tumor formation. Oncogene. 2007;26:7204–12. 17496925 10.1038/sj.onc.1210527

[66] Foulkes WD, Metcalfe K, Sun P, Hanna WM, Lynch HT, Ghadirian P, et al. Estrogen receptor status in BRCA1- and BRCA2-related breast cancer: the influence of age, grade, and histological type. Clin Cancer Res. 2004;10:2029–34. 15041722 10.1158/1078-0432.ccr-03-1061

[67] Kim H, Heo K, Kim JH, Kim K, Choi J, An W. Requirement of histone methyltransferase SMYD3 for estrogen receptor-mediated transcription. J Biol Chem. 2009;284:19867–77. 19509295 10.1074/jbc.M109.021485 PMC2740412

[68] Fang Y, Shen X. Ubiquitin carboxyl-terminal hydrolases: involvement in cancer progression and clinical implications. Cancer Metastasis Rev. 2017;36:669–82. 29080080 10.1007/s10555-017-9702-0

[69] Rajajeyabalachandran G, Kumar S, Murugesan T, Ekambaram S, Padmavathy R, Jegatheesan SK, et al. Therapeutical potential of deregulated lysine methyltransferase SMYD3 as a safe target for novel anticancer agents. Expert Opin Ther Targets. 2017;21:145–57. 28019723 10.1080/14728222.2017.1272580

[70] Huang L, Xu AM. SET and MYND domain containing protein 3 in cancer. Am J Transl Res. 2017;9:1–14. 28123630 PMC5250700

[71] Xu Z, Wang W, Deng CX, Man YG. Aberrant p63 and WT-1 expression in myoepithelial cells of pregnancy-associated breast cancer: implications for tumor aggressiveness and invasiveness. Int J Biol Sci. 2009;5:82–96. 19173015 10.7150/ijbs.5.82 PMC2631157

[72] O’Brien J, Lyons T, Monks J, Lucia MS, Wilson RS, Hines L, et al. Alternatively activated macrophages and collagen remodeling characterize the postpartum involuting mammary gland across species. Am J Pathol. 2010;176:1241–55. 20110414 10.2353/ajpath.2010.090735 PMC2832146

[73] Xiao C, Sharp JA, Kawahara M, Davalos AR, Difilippantonio MJ, Hu Y, et al. The XIST noncoding RNA functions independently of BRCA1 in X inactivation. Cell. 2007;128:977–89. 17350580 10.1016/j.cell.2007.01.034

[74] Kim D, Langmead B, Salzberg SL. HISAT: a fast spliced aligner with low memory requirements. Nat Methods. 2015;12:357–60. 25751142 10.1038/nmeth.3317 PMC4655817

[75] Liao Y, Smyth GK, Shi W. featureCounts: an efficient general purpose program for assigning sequence reads to genomic features. Bioinformatics. 2014;30:923–30. 24227677 10.1093/bioinformatics/btt656

[76] Love MI, Huber W, Anders S. Moderated estimation of fold change and dispersion for RNA-seq data with DESeq2. Genome Biol. 2014;15:550. 25516281 10.1186/s13059-014-0550-8 PMC4302049

[77] Wu T, Hu E, Xu S, Chen M, Guo P, Dai Z, et al. clusterProfiler 4.0: A universal enrichment tool for interpreting omics data. Innovation. 2021;2:100141. 34557778 10.1016/j.xinn.2021.100141 PMC8454663

[78] Nicol JW, Helt GA, Blanchard SG Jr, Raja A, Loraine AE. The Integrated Genome Browser: free software for distribution and exploration of genome-scale datasets. Bioinformatics. 2009;25:2730–1. 19654113 10.1093/bioinformatics/btp472 PMC2759552

[79] Shen L, Shao NY, Liu X, Maze I, Feng J, Nestler EJ. diffReps: detecting differential chromatin modification sites from ChIP-seq data with biological replicates. PLoS ONE. 2013;8:e65598. 23762400 10.1371/journal.pone.0065598 PMC3677880

