# Supplementary material for: Oncogenic activation of SMYD3-SHCBP1 promotes breast cancer development and is coupled with resistance to immune therapy
Source: Cell Death Dis. 2025 Mar 29;16(1):220. doi: 10.1038/s41419-025-07570-8 (PMC11954966; doi:10.1038/s41419-025-07570-8)
Supplement: Supplementary file 1 — Supplementary Figures [file 41419_2025_7570_MOESM1_ESM.pdf]

Oncogenic Activation of SMYD3-SHCBP1 Promotes Breast Cancer Development and is Coupled with Resistance to Immune Therapy

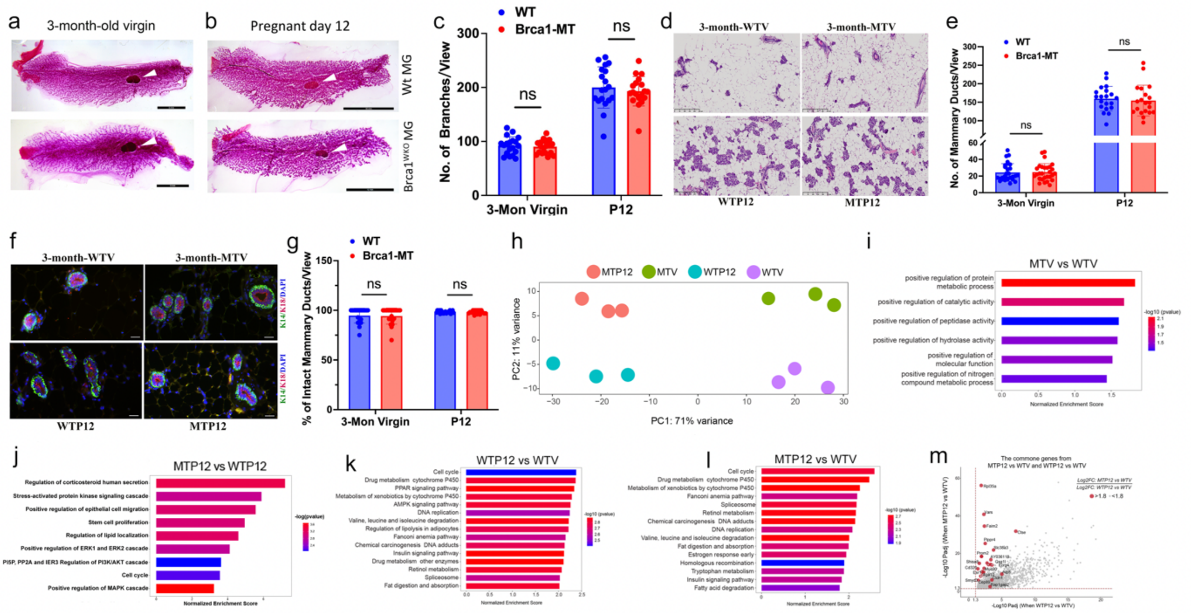

Supplementary Fig. 1 Analysis of gene expressions of mammary epithelial cells in WT and Brca1^{MKO} virgin and P12 mice.

(a-c) Representative images of wholemount mammary glands from 3-month-old virgin (a) and at pregnant day 12 (P12) (b) in both WT and Brca1^{MKO} mice imaged by Leica M165FC dissecting microscope, and the quantifications of ducts in each genotype of mice (c). The 19 images were used in all four groups: WTVs with 1753 branches, MTVs with 1715 branches, WTP12 with 3798 branches, MTP12 with 3667 branches (d). (n=4 mice/group). White arrowheads point to lymph node.

(d, e) Representative images of H & E sections from 3-month-old WTV, MTV, WTP12, MTP12 mice (d) and quantifications of mammary ducts (e). The 28 images were used for virgin mice that contain 682 and 691 ducts in WTV and MTV, respectively. 20 images were used for P12 mice that contain 3191 and 3091 ducts in WTP12 and MTP12, respectively, (n=6 mice/group).

(f, g) Representative mammary glands images of 3-month-WTV, 3-month-MTV, WTP12, and MTP12 staining with antibodies of K14 and K18 (f) and quantifications with bar graph of mammary gland ducts with “intact” structure or “non-intact” structure (g). The 20 images were used in each group (n=6 mice/group).

(h) Sample quality control (QC) Status visualized by PCA plot for the 12 samples used form bulk RNA sequences.

(i-l) Enrichment pathways from the comparison of up regulated genes in MTV vs WTV (i), MTP12 vs WTP12 (j), WTP12 vs WTV (k), and MTP12 vs WTV (l) were generated by GSEA analysis with criteria of NES score >1.0 and p value < 0.05. Top 15 pathways are listed in WTP12 vs WTV and WTP12 vs WTV. In MTV vs WTV, there are only 6 enriched pathways have the p<0.05.

(m) Scatter plots visualized by using R pacakge “EnhancedVolcano” tool [Blighe, K, S Rana, and M Lewis. 2018. The genes with Log2FC>0.5 and q-value<0.05 (-log10>1.3) are considered as statistically significant. The expression ratio of top 20 candidate genes based on their Log2FC between MTP12 vs WTV to WTP12 vs WTV.

The bigger red color dots are the one with the ratio is bigger than 1.8-fold and the small grey dots are the one with ration is smaller than 1.8-fold. The calculation formula is on the right upper corner.

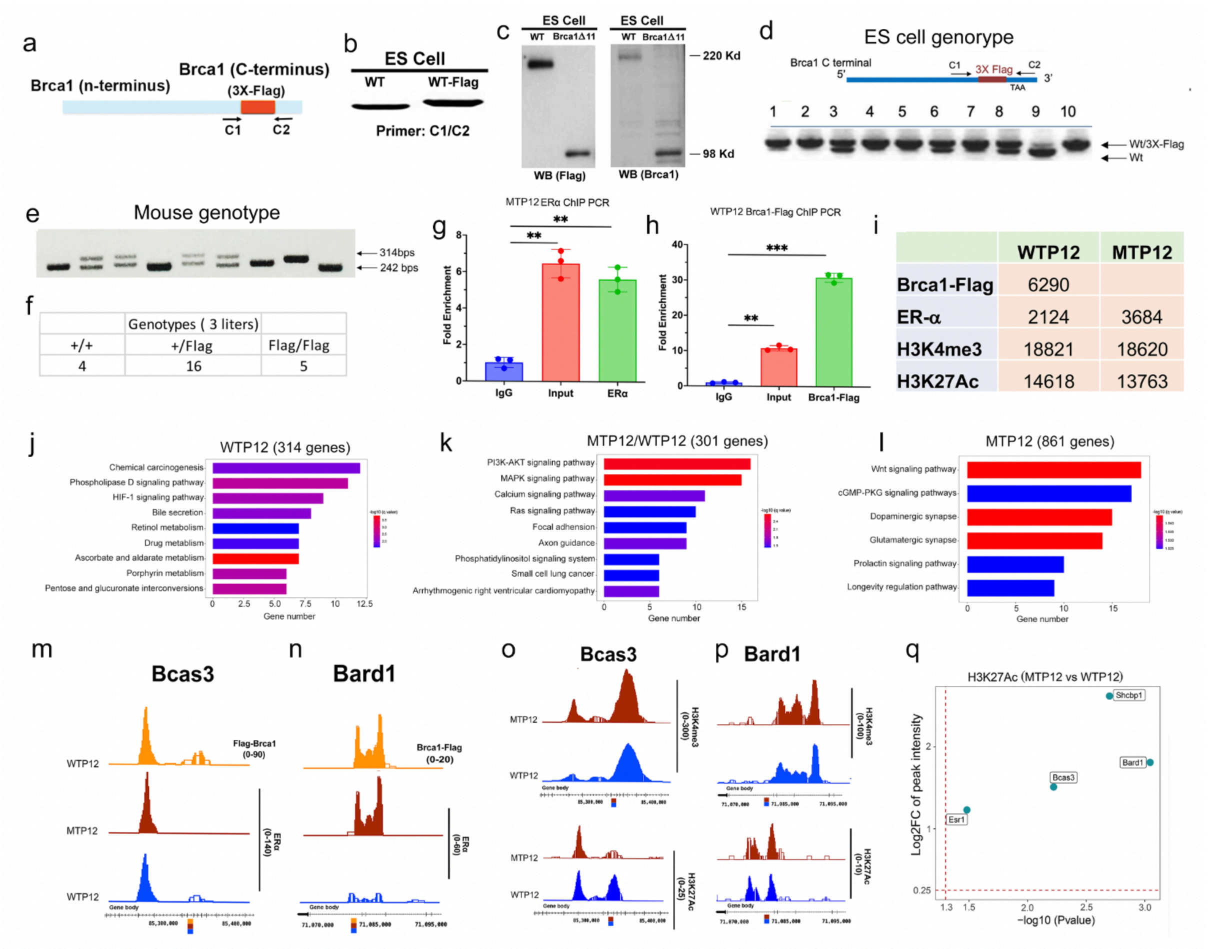

Supplementary Fig. 2 Analysis of ChIP-seq with mammary epithelial cells in WTP12 and MTP12 mice.
(a) The structure of the mouse Brca1 gene with 3X Flag inserted at the C-terminal end before the stop codon of the Brca1 gene.
(b) Genotypes of WT ES cells and WT-Flag ES cells by PCR with primers of C1 and C2 flanked the 3X-Flag sequence.
(c) Brca1 protein levels detected by Flag antibody (left panel) and a Brca1 antibody (right panel) by Western blots.
(d) The representative 3X-Flag targeted ES clones (314 bps) and Wt clones (242 bps) with primers of flanking 3X-Flag sequence by PCR.
(e, f) Representative gel images from 3 liters of heterozygotes (+/Flag) mice mating (e) and the distributions of three different genotypes of WT, +/Flag and Flag/Flag (i) mice (f). The Flag/Flag mice were developmentally normal up to 18 months of age.
(g, h) The relative fold enrichments by ChIP-qPCR against the antibodies of ERα (g) in MTP12 cells and Flag-Brca1 (h) in WTP12.

(i) The number of genes detected by ChIP-seq with antibodies of Flag for Brca1 in WTP12, ERα, H3K4me3, and H3K27Ac in WTP12 and MTP12 (n=6 mice/group).

(j-l) KEGG pathway analysis with 314 genes that were pull down by antibodies of Flag-Brca1, ERα, H3K4me3, and H3K27Ac in WTP12 only (j), 301 common genes pull down in both WTP12 and MTP12 (k), and 861 genes pull down in MTP12 cell only (l) from Fig.1k.The bar plots represent the enriched pathways with q-value < 0.05 in supplementary data 6.

(m-p) Visualization of differential ChIP binding peaks on gene body of Bcas3 (m) and Bard1 (n) genes against Brca1-Flag (orange color) in WTP12 cells, ERα (burgundy color) in MTP12 and ERα (blue color) in WTP12. Differential ChIP binding peaks on gene bodies of Bcas3 (o) and Bard1 (p) genes against antibodies of H3K4me3 in MTP12 (burgundy color) and in WTP12 (blue color) and H3K27ac in MTP12 (burgundy color) and in WTP12 (blue color).

(q) Visualization of differential ChIP binding intensities by H3K27Ac enzyme of Esr1, Bcas3, Bard1, and Shcbp1 genes in MTP12 vs WTP12 mice. P value < 0.05, that is, -log10(P value) > 1.3 and log2 (fold change) > 0.25.

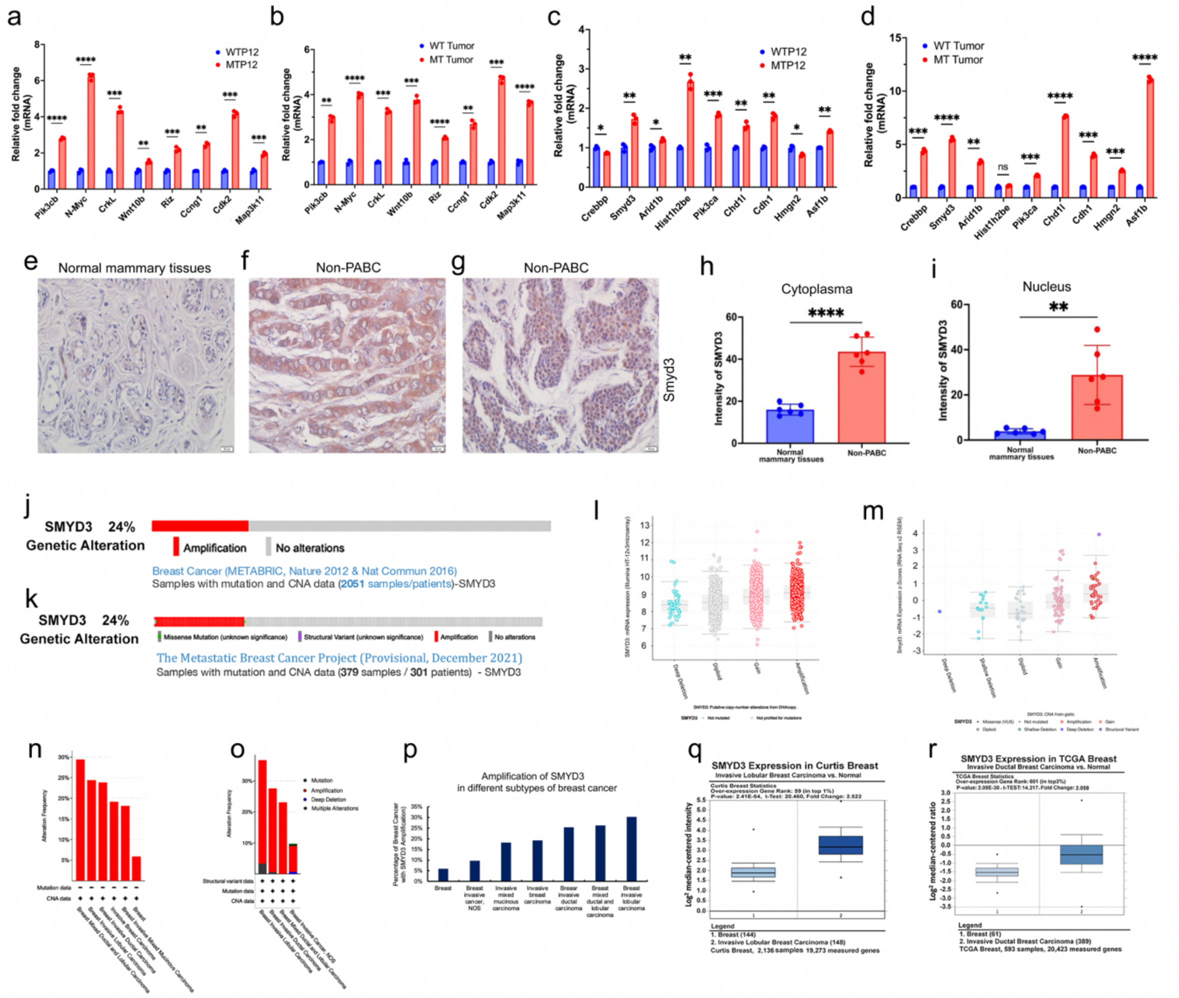

Supplementary Fig. 3 SMYD3 expression is elevated in different types of breast cancer.

(a, b) Expressions of Smyd3 downstream genes in P12 MG tissues (a) and tumor tissues (b) of both WT and Brca1^{MKO} mice by qPCR. (n=3 mice/group).

(c, d) Expressions of candidate genes in P12 MG tissues (c) and tumor tissues (d) in WT and Brca1^{MKO} mice, as determined by qPCR. (n=3 mice from each group).

(e-i) Protein levels of SMYD3 on the normal mammary control tissues (e), non-PABC tumor tissues in cytoplasm (f), non-PABC tumor tissues in nucleus (g) by IHC staining. The quantification of SMYD3 protein levels in cytoplasm (h) and in nucleus (i) by Image J (n=6 samples/group).

(j, k) Percent of SMYD3 with amplification of CNV in breast cancer patients from Metabric dataset, Nature 2012 & Nature Communication 2016 (j) and the Metastatic Breast Cancer Project (Provisional, April 2021) (k). (https://www.cbioportal.org).

(l, m) Expression of SMYD3 in Metabric dataset, Nature 2012-2016 & Nature Commun 2016 (l) and Metastatic Breast Cancer Project (Provisional, April 2021) (m) from cBioportal data sets searched with SMYD3 expression.

(n, o) The SMYD3 expression in different subtypes of breast cancer patients with invasive lobular breast carcinoma (n) and invasive ductal carcinoma (o) from the TCGA dataset.

(p) Levels of SMYD3 expression (obtained from the cBioportal database) are increased in all different subtypes of breast cancer, with the highest in the invasive lobular breast cancer.

(q-r) The SMYD3 expressions in invasive lobular breast cancer (q), ductal breast invasive carcinoma (r) from Curtis Breast Cancer Dataset and TCGA dataset.

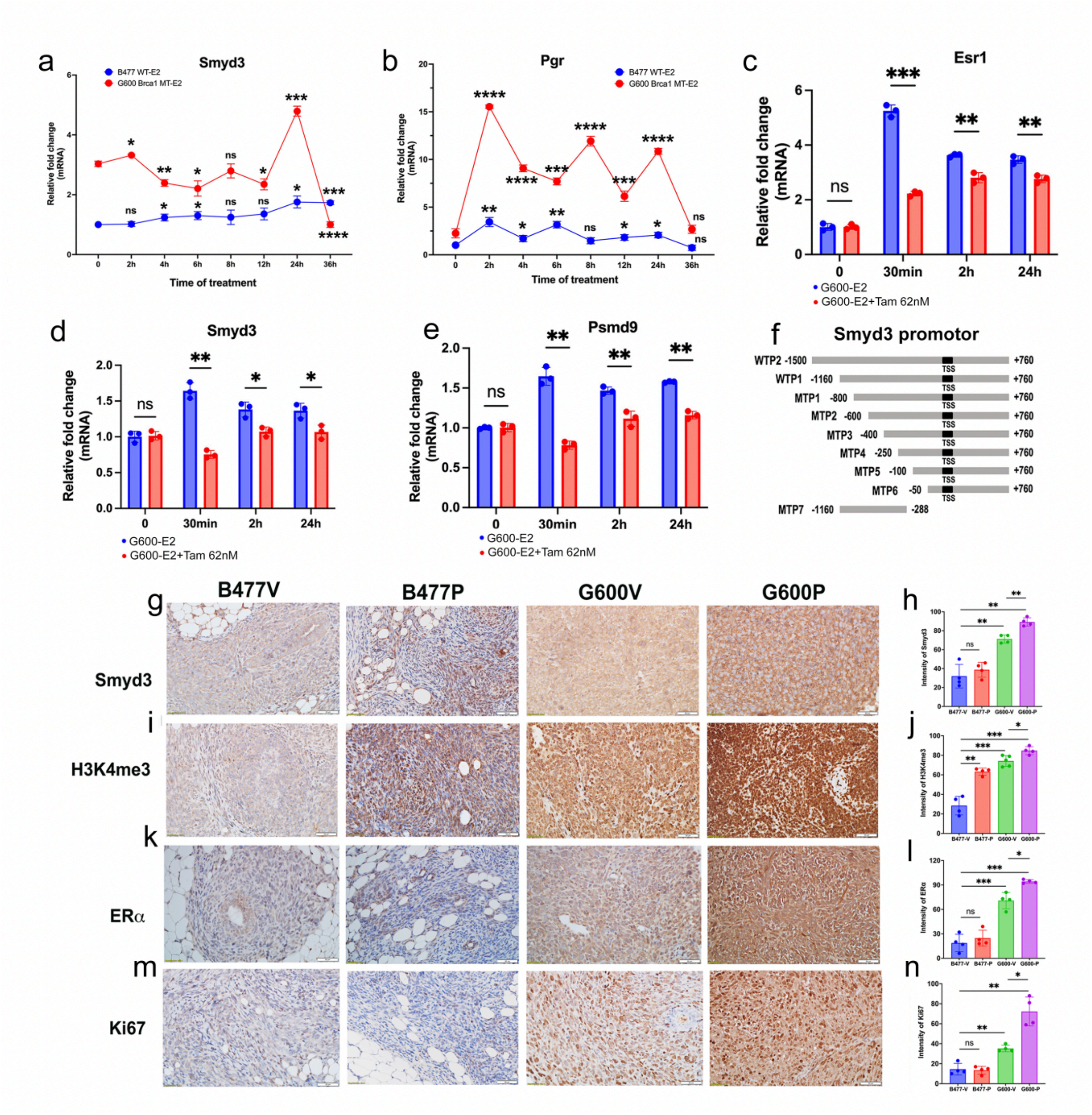

Supplementary Fig. 4 ERα positively potentiates oncogenic signals.

(a, b) The expression patterns of some oncogenic genes in B477 and G600 mammary epithelial cell lines after E2 treatment for 36 hours, including Smyd3 (a), and Pgr (b).

(c-e) The expression patterns of Esr1(c), Smyd3 (d), and Psmd9 (e) treated with E2 (100nM) or together with tamoxifen (62nM) at 0, 30 min., 2 hours, and 24 hours in G600 cell.

(f) The design of Smyd3 promoters with serial deletions from -1500 bps to +760 bps and the sequence was downloaded from the EPD promoter database (https://epd.epfl.ch//index.php).

(g-n) Representative images of IHC staining and quantification of protein levels against antibodies of Smyd3 (g, h) H3K4me3 (i, j), ERα (k, l), and Ki67 (m, n) in nude virgin mice and mice experienced full-term pregnancy implanted with WT B477 cells and Brca1-MT G600 cells. Scale bar is 50 μm.

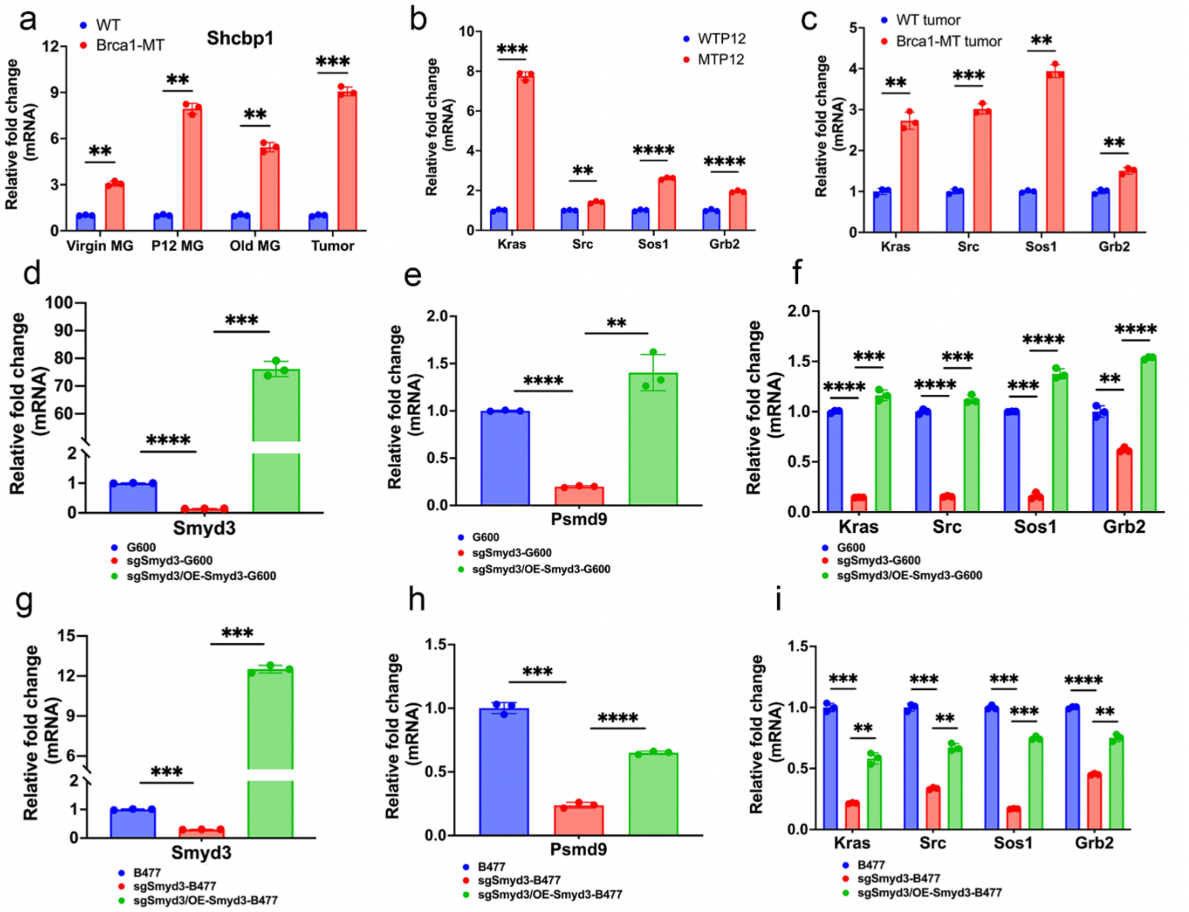

Supplementary Fig. 5 Elevated oncogenic expression induced by Smyd3.

(a-c) The relative Shcbp1 mRNA expression of mammary tissues from WTP12 and MTP12 mice and tumor tissues (a), the expression of Kras, Src, Sos1, and Grb2 in WTP12 and MTP12 mammary tissues (b), and in WT and Brca1-MT tumor tissues (c).

(d-f) The expression of Smyd3 (d), Psmd9 (e), Kras, Src, Sos1, and Grb2 (f) in sgSmyd3-G600 cells with the OE-Smyd3.

(g-i) The expression of Smyd3 (g), Psmd9 (h), Kras, Src, Sos1, and Grb2 (i) in sgSmyd3-B477 cells with the OE-Smyd3.

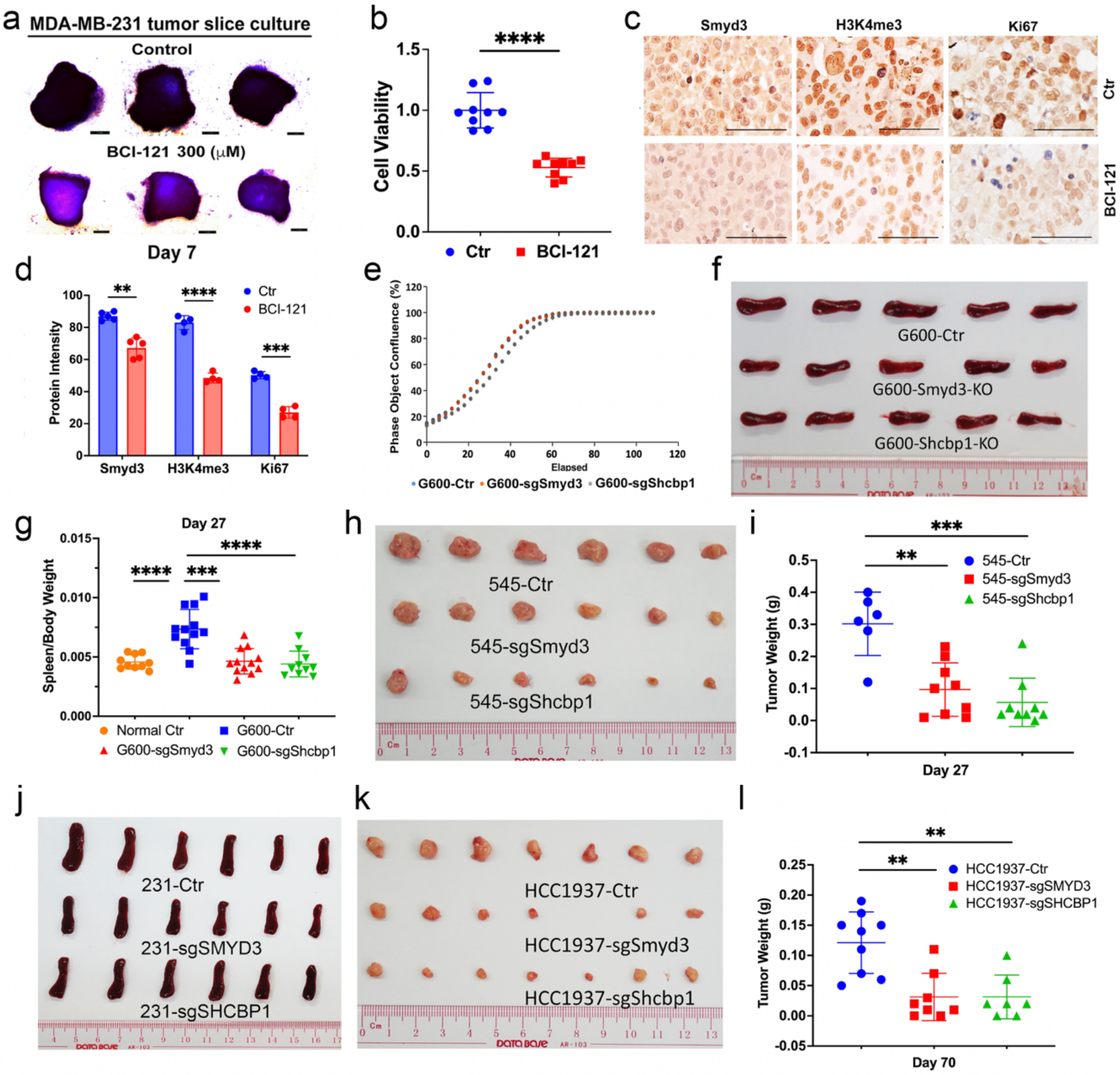

Supplementary Fig. 6 Disruption of the oncogenic signals of either SMYD3 or SHCBP1 reverses the splenomegaly phenotype.

(a, b) Representative viability images of the slices from 231 tumors without (control), or with the treatment of BCI-121 at 300 μM assayed by MTT (a) and the quantifications (b) in (a) (n=3 mice/group).

(c, d) The IHC staining with antibodies of Smyd3, H3K4me3, and Ki67 (c), and quantifications (d) of protein intensities from the same cohort of 231 tumor slices in (a-b) (n=3 mice /group). Scale bar is 50 μm.

(e) The cell growth curves of G600 parental, sgSmyd3-G600, and sgShcbp1-G600 cells were measured by IncuCyte.

(f, g) Representative images of spleens from the nude mice with the implantation of parental G600, sgSmyd3-G600, and sgShcbp1-G600 cells at 2X10^5 cells per mammary fat pad of nude mice (f) and quantification (g) in (f) (n=10-12 mice/group).

(h, i) The tumor image (h) and tumor weight (i) from FVB mice implanted with 545 parental, sgSmyd3-545, and sgShcbp1-545 cells at 5X10^6 cells per mammary fat pad for 27 days (n= 6-9 mice/group).

(j) Representative images of the spleen from the nude mice with the implantation of parental MDA-MB-231 (231), sgSMYD3-231, and sgSHCBP1-231 cells at 2X10^6 cells per mammary fat pad for 70 days (n=12 mice/group).

(k, l) Tumor images from the nude mice with the fat pad implantation of parental HCC1937, sgSMYD3-1937, and sgSHCBP1-1937 cells at 5X10^{6} cells for 70 days (k) and the tumor weight plot (l) in (k) (n=7-9 mice/group).

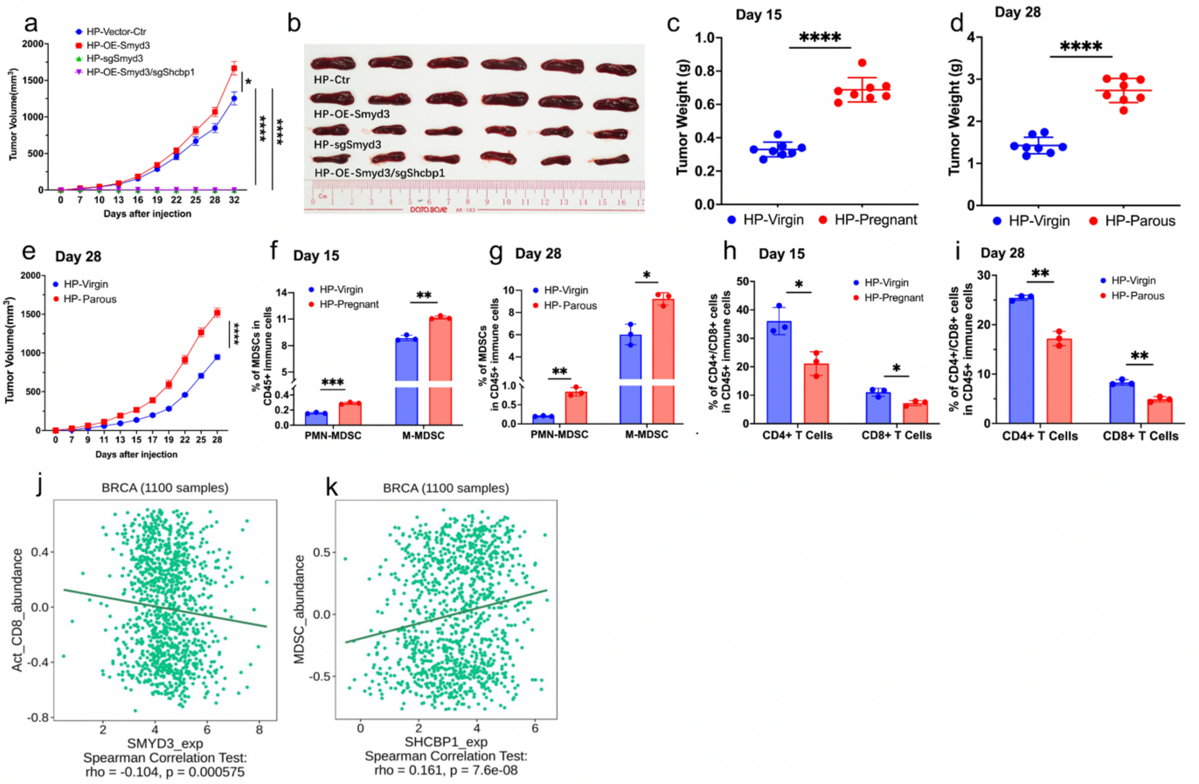

Supplementary Fig. 7 Smyd3-Shcbp1 signaling shapes TME in pregnant mice and involution mice.
(a) Tumor volumes from FVB mice implanted with HP5712 parental, OE-Smyd3-HP5712, sgSmyd3-HP5712, and OE-Smyd3/sgShcbp1-HP5712 cells at 1X10^{6} cells per mammary fat pad for 32 days (n=8 mice/group).
(b) Representative spleen images from the same cohort of mice in (Fig. 6c).
(c) The plot represents tumor growth by weight on day 15 after implantation of HP cells in FVB virgin and pregnant mice, respectively. (n=8 tumors/each group).
(d) The plot represents tumor growth by weight on day 28 (7 days after newborn delivery) after implantation of HP cells in FVB virgin or pregnant mice, respectively. (n=8 tumors/each group).
(e) Tumor volume from FVB virgin mice and pregnant mice with implantation of HP5712 cells in fat pad for 28 days. The tumors were harvested on day 7 after birth in parous group (n=8 mice/group).
(f, g) MDSC populations in both virgin and pregnant mice at day 15 (f) and day 28 (g) (7 days after newborn delivery) with implantations of HP cells to FVB mice by CyTOF analysis (n=3 mice/each group)
(h, i) CD8+ and CD4+T cell populations in both virgin and pregnant mice at day 15 (h) and day 28 (i) (7 days after newborn delivery) with implantations of HP cells to FVB mice by CyTOF analysis (n=3 mice/each group)
(j, k) Correlations between SMYD3 and the abundance of activated CD8 T cells in TCGA BRCA data from the TISIDB tool (j) and between SHCBP1 and MDSC's abundance in TCGA BRCA data from the TISIDB tool (k).
TISIDB: an integrated repository portal for tumor-system interactions. Bioinformatics. 2019; btz21immune

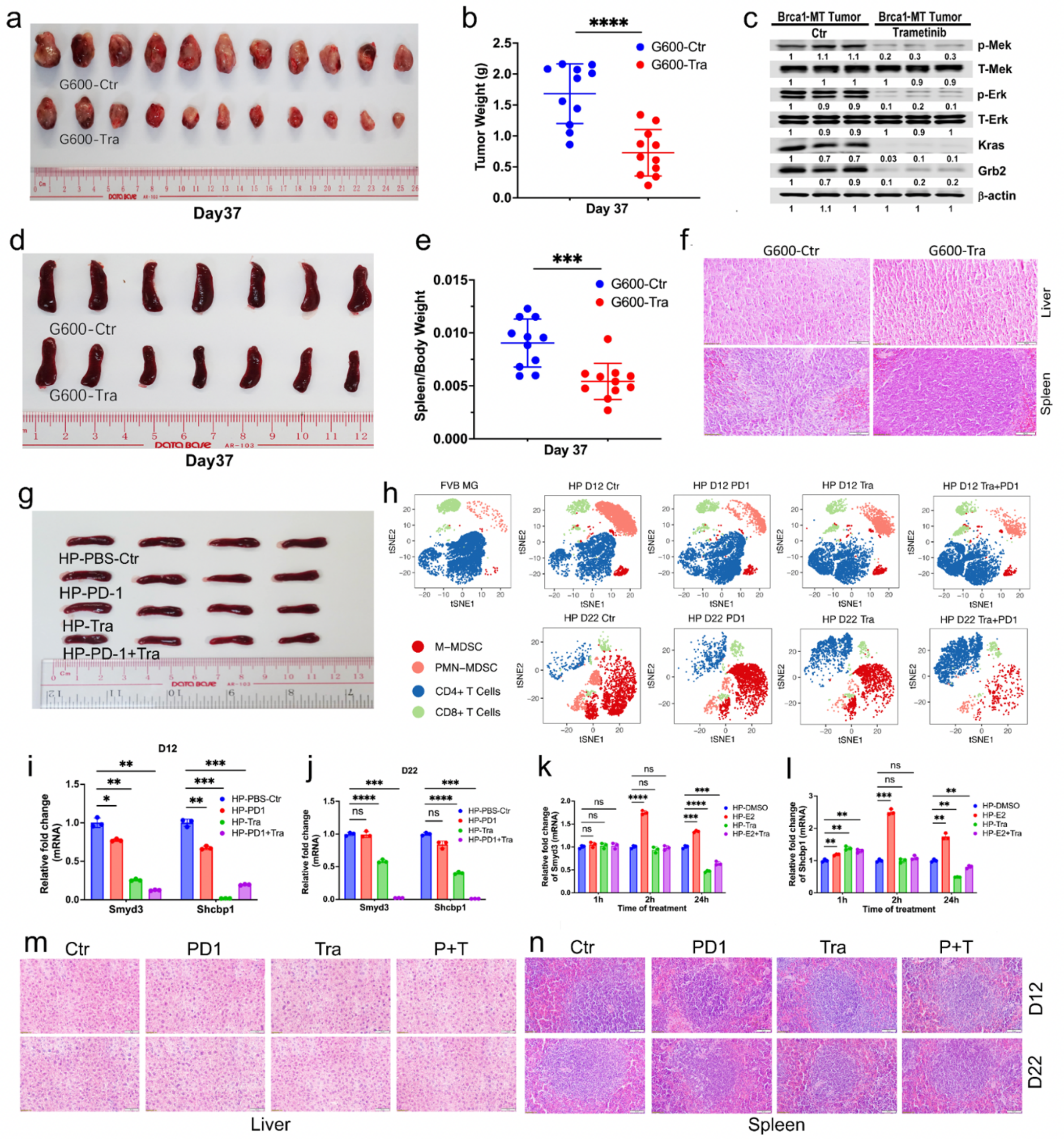

Supplementary Fig. 8 Inhibition of the oncogenic action of Smyd3 reverse TIME and tumor growth. (a, b) Representative tumor images (a) and tumor weight plot (b) without or with trametinib (Tra) treatment in nude mice. The $1X10^5$ of G600 cells per fat pad were implanted and the Tra was delivered through gavage at 0.5mg/kg every other day starting when the tumor reached 150-200 mm^3 (7 days later after implantation) for 37 days (n=11 mice/group).

(c) Protein levels of pMek, pErk, Kras and Grb2 of breast tumor tissues from the mice in (a-b) treated with Tra by Western blots.

(d, e) Images of spleen (d) and quantifications (e) in (d) from the same cohort of mice in (a-b).

(f) The H&E staining of liver and spleen, respectively from the same cohort of mice in (a). Scale bar is 50µm.

(g) Representative spleen images from FVB mice implanted with HP5712 cells and treatment with PBS, αPD1, Tra, and αPD1+Tra.

(h) tSNE visualized immune cells from normal FVB mouse mammary glands (FVB MG), tumors implanted with HP5712 cells at $1X10^6$ cells per mammary fat pad, and treatment with PBS, αPD1, Tra, and PD1+Tra from FVB mice at D12 and D22 (n = 3 mice/group).

(i, j) Expressions of Smyd3 and Shcbp1 in mammary tissues from the HP cell implanted mice treated with αPD1, Tra, and PD1+Tra from FVB mice at D12 (i) and D22 (j) as determined by qPCR (n = 3 mice/group).

(k, l) Expressions of Smyd3 (k) and Shcbp1 (l) of HP cells with the treatment of E2, Tra, and E2 together with Tra at 1 hour, 2 hours, and 24 hours as determined by qPCR.

(m, n) H&E staining of the liver (m) and spleen (n) from FVB mice implanted with HP5712 cells and treatment with PBS, αPD1, Tra, and αPD1+Tra at D12 and D22. Scale bar is 50μm.

Error bars show mean ± SD. The Welch t-test or nonparametric test was used to calculate significance. $*P < 0.05$, $**P < 0.01$, $***P < 0.0001$, $****\ P < 0.00001$.
