# Supplementary material for: Oncogenic activation of SMYD3-SHCBP1 promotes breast cancer development and is coupled with resistance to immune therapy
Source: Cell Death Dis. 2025 Mar 29;16(1):220. doi: 10.1038/s41419-025-07570-8 (PMC11954966; doi:10.1038/s41419-025-07570-8)
Supplement: Supplementary file 2 — Legends for Supplementary data 1-8 [file 41419_2025_7570_MOESM2_ESM.docx]

Supplementary data 1: Bulk RNA sequences from both virgin mice and pregnant mice at P12.

The RNA-sequence data from different genotypes of mice, including WT virgin mice (WTV, n=6 mice/group), Brca1^MKO^ virgin mice (MTV, n=6 mice/group), WT mice at pregnant day 12 (WTP12, n=6 mice/group), Brca1^MKO^ mice at pregnant day 12 (MTP12, n=6 mice/group) mice. Differentially expressed genes were called through a negative binomial generalized linear model fitted to median of ratios normalized values, as recommended in the DESeq2 R package manual from Genome Biology, 2014, 15(12): 1-21. Differentially expressed genes were determined by cutoffs of Benjamini-Hochberg adjusted p < 0.05 and log2Fold Changes at least 0.5 in absolute value.

Supplementary data 2: DE Gene lists of MTV vs WTV WTP12 vs WTV, MTP12 vs WTV, and MTV vs WTV.

1753 up regulated genes and 2139 down regulated genes are comparison from WTP12 vs WTV group, 1940 up regulated genes and 2226 down regulated genes are from MTP12 vs WTV, 233 up regulated genes and 363 down regulated genes are from MTV vs WTV group. The cut off criteria is: if the Log2FC < -0.5 with adjusted p value < 0.05, we classified as down regulated genes. If the Log2FC > 0.5 with adjusted p-value < 0.05, we classified as up regulated genes.

Supplementary data 3: Heatmap with comparisons of MTP12 and WTP12 to WTV.

The upregulated genes in WTP12 and MTP12 with scaled mean expression from normalized counts of each gene are combined first with removed duplication gene names and then extract out the *same* gene list from WTV group to generate the heatmap by using the R package "pheatmap".

Supplementary data 4. Gene Set Enrichment Analysis (GSEA) with bulk RNA sequences.

(Sheet 1) Enriched pathways with NES score >1.0 and P value < 0.05 from the comparison between MTV vs WTV. (Sheet 2) Top 15 enriched pathways between WTP12 vs WTV with NES score >1.0 and p value < 0.05. (Sheet 3) Top 15 enriched pathways between MTP12 vs WTV with NES score > 1.0 and p value < 0.05. With calculation tool for GSEA analysis: the R package "clusterProfiler" calculation.

Supplementary data 5. Gene lists from ChIP sequence by antibodies of H3K4me3, H3K27AC, ERα, Flag.

6290 genes are identified by Flag antibody in WTP12, 2124 genes are identified by ERα in WTP12, 3648 genes are identified by ERα in MTP12, 18821 genes are identified by H3K4me3 antibody in WTP12 mice, 18620 genes are identified by H3K4me3 antibody in MTP12 mice. 14618 genes are identified by H3K27Ac antibody in WTP12 mice. 13763 genes are identified by H3K27Ac antibody in MTP12 mice. MACS2 tools were used for peak calling and the q-value cutoff for peak detection was set to 0.05.

Supplementary data 6. KEGG pathway analysis from 314, 301, and 861 gene lists identified from ChIP sequences.

314 gene list is WTP12 only, 310 gene list contain the genes that can be found in both WTP12 and MTP12, and 861 gene list is in MTP12 only. The enriched oncogenic pathways were listed in this table with calculation of BgRatio, p-value, p-adjust value, q-value, GeneID, and Gene count using R package 'clusterProfiler' (v4.7.1) with KEGG database (doi: 10.1016/j.xinn.2021.100141).

Supplementary data 7. Comparison of chromatin remodeling genes to 1940 gene list from bulk-RNA-seq and 1162 genes from ChIP-seq in MTP12

The comparison of 1940 genes from MTP12 vs WTV gene lists by RNA sequences and 1162 gene list from ChIP-seq in MTP12.

Comparison of 176 chromatin remodeling gene list was obtained from protein and DNA binding dataset (GO:0065004) to the 131 common gene lists from bulk-RNA-seq and ChIP-seq.

Supplementary data 8. KEGG pathways enrichment analysis from 1162 genes in MTP12 detected by ChIP sequences.

The enriched oncogenic pathways were listed in this table with calculation of BgRatio, p-value, p-adjust value, q-value, GeneID, and Gene count using R package 'clusterProfiler' (v4.7.1) with KEGG database (doi: 10.1016/j.xinn.2021.100141).
