# Supplementary material for: Oncogenic activation of SMYD3-SHCBP1 promotes breast cancer development and is coupled with resistance to immune therapy
Source: Cell Death Dis. 2025 Mar 29;16(1):220. doi: 10.1038/s41419-025-07570-8 (PMC11954966; doi:10.1038/s41419-025-07570-8)
Supplement: Supplementary file 11 — Full length western blots [file 41419_2025_7570_MOESM11_ESM.docx]

**Full length western blots**

**Fig 2f Fig 2g**

**
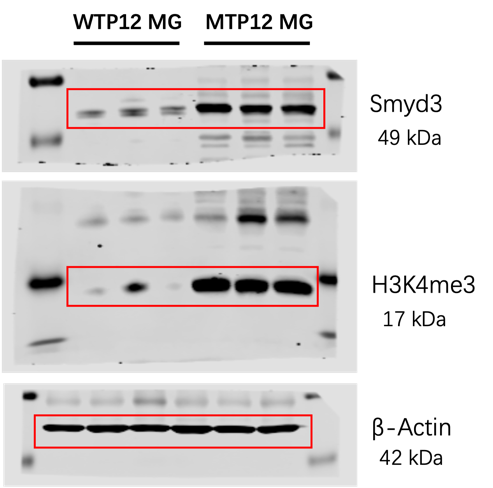

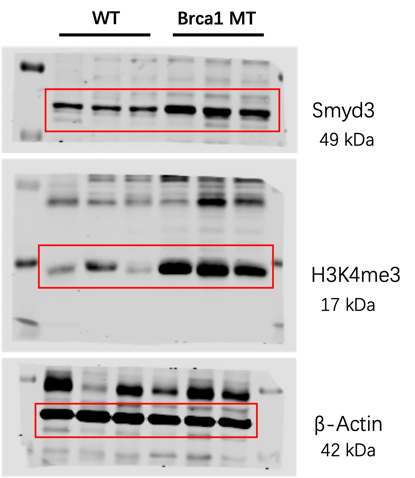
**

**Fig 3k**

**
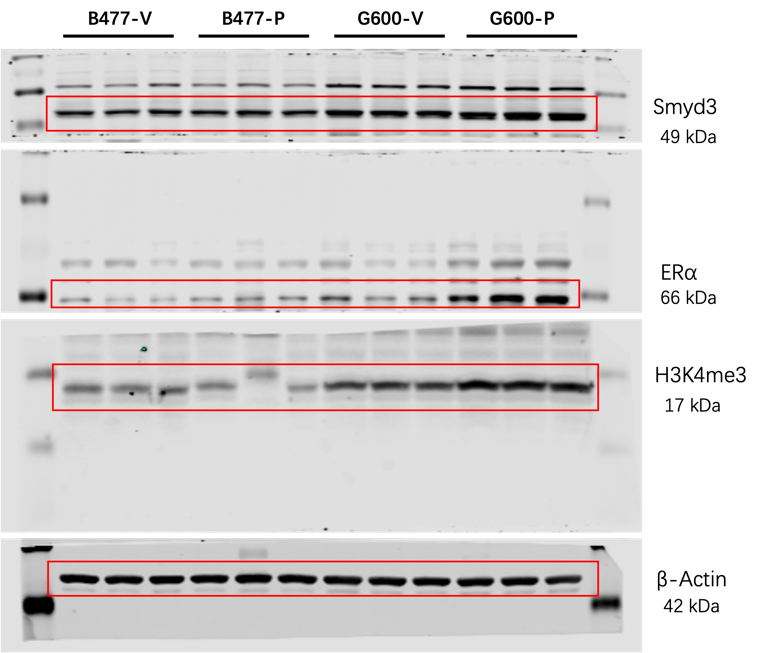
**

**Fig 4e Fig 4g**

**
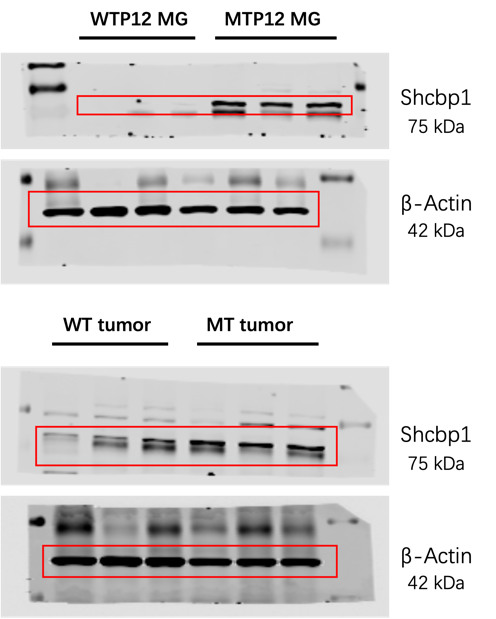

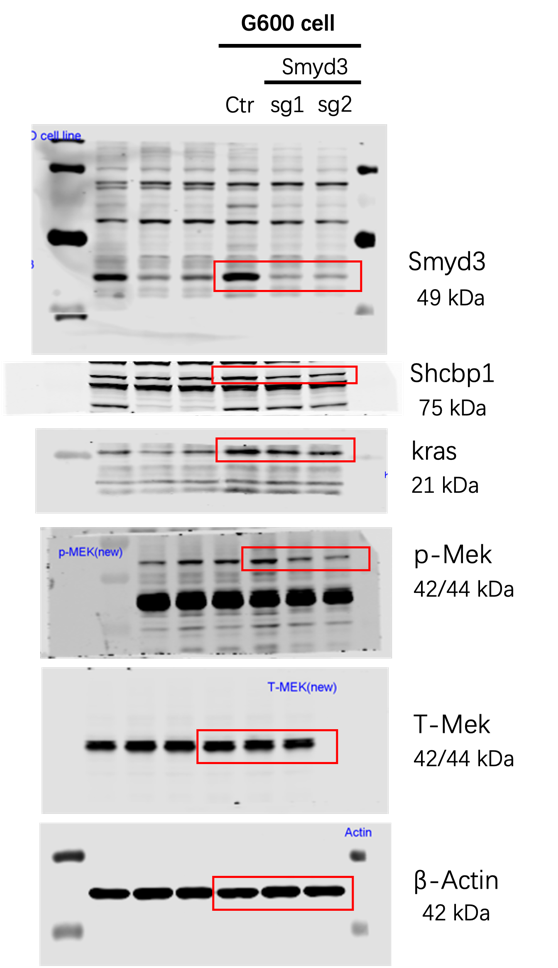
**

**Fig 4h Fig 4i**

**
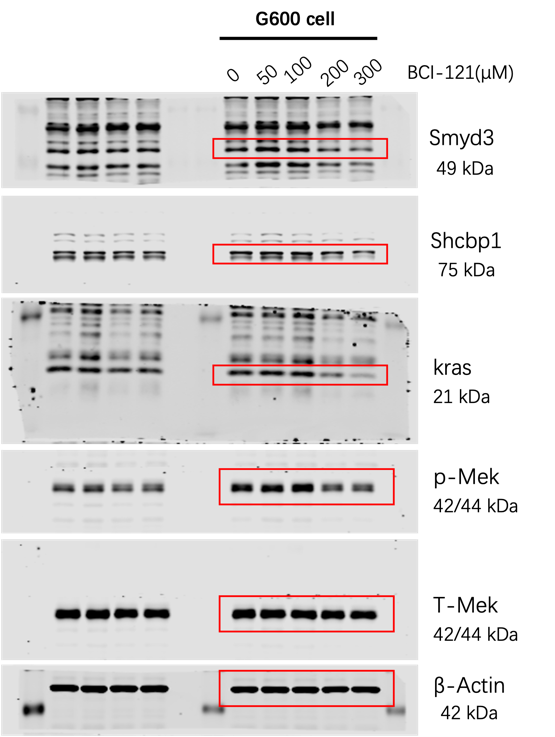

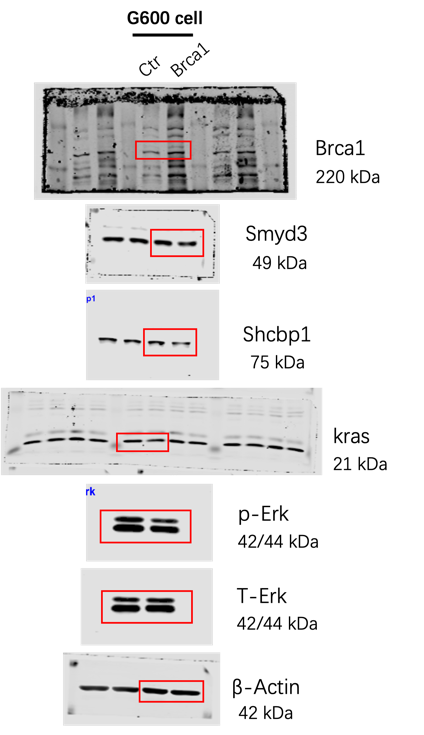
**

**Fig 4j Fig 4k**

**
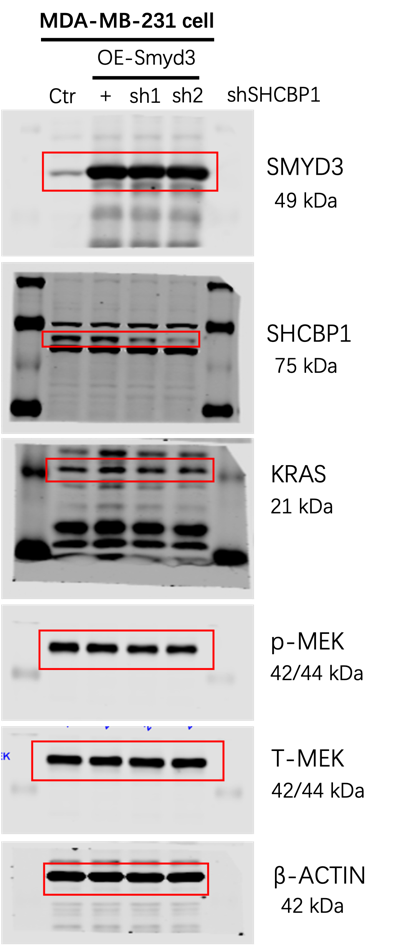

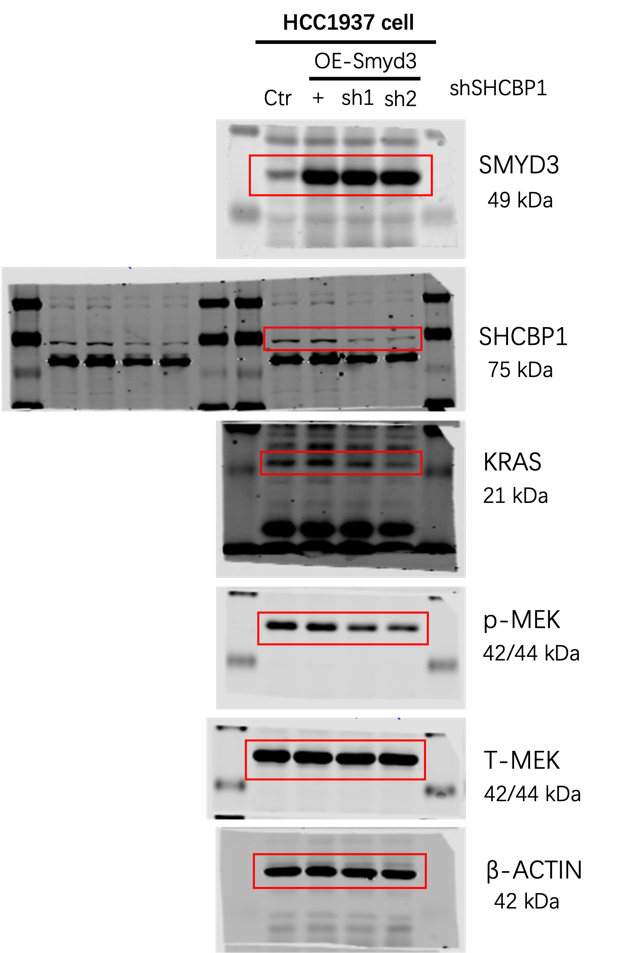
**

**Fig 5i Fig 5j**

**
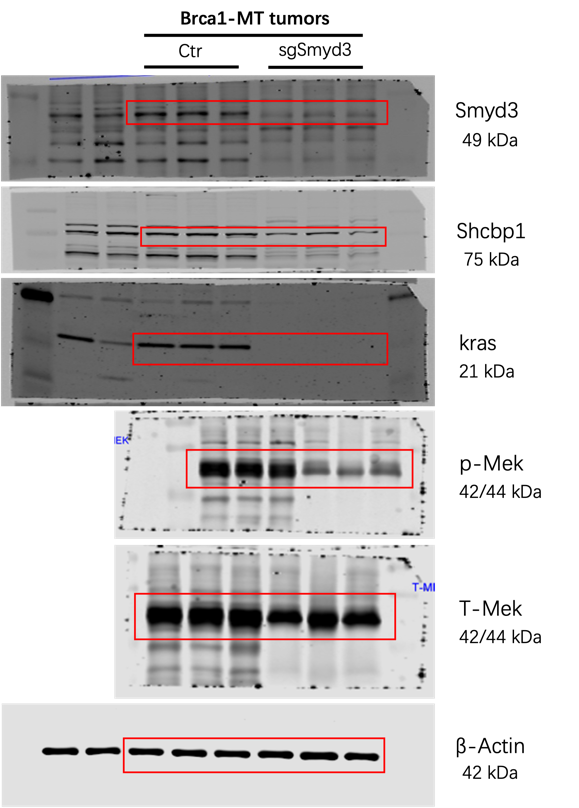

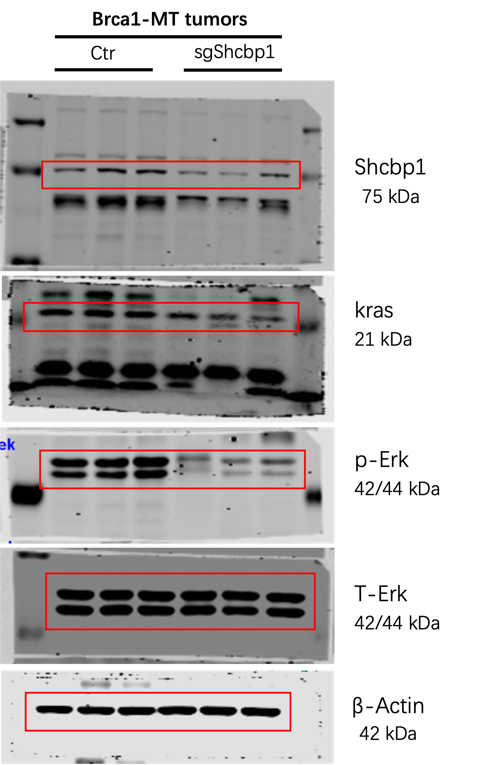
**

**Fig 5o Fig 5p**

**
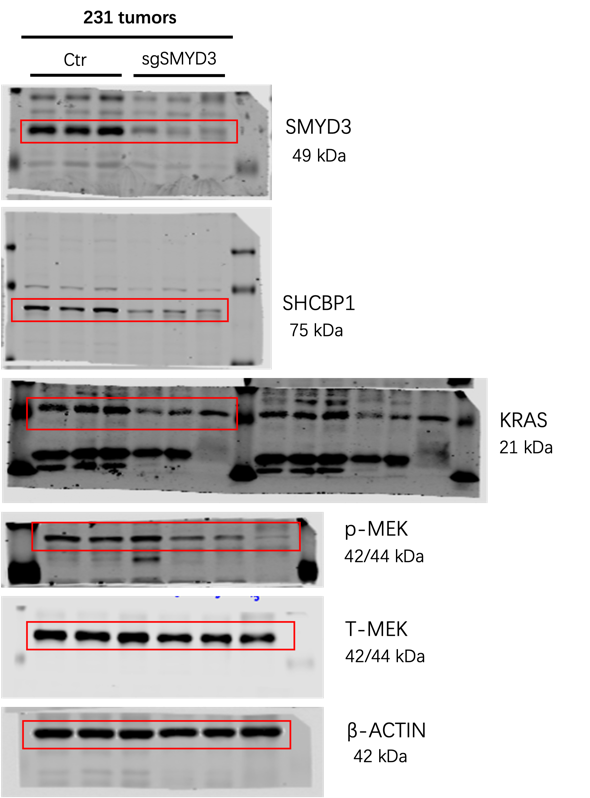

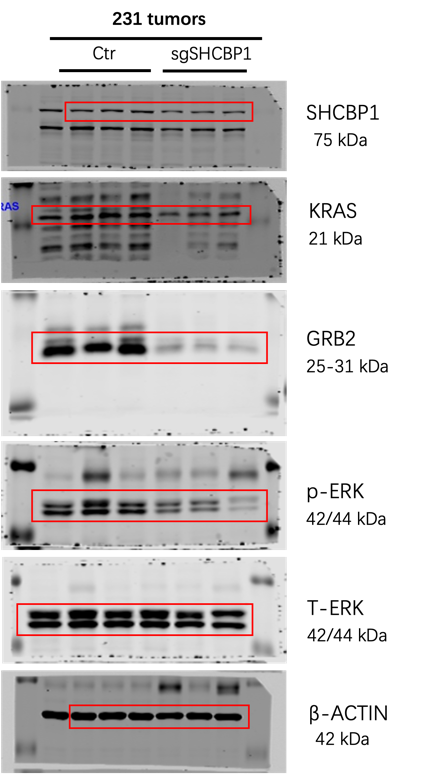
**

**Fig 6a Fig 6b**

**
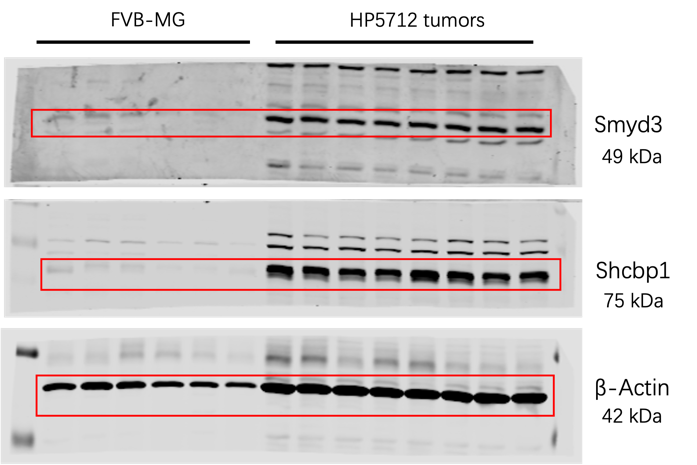

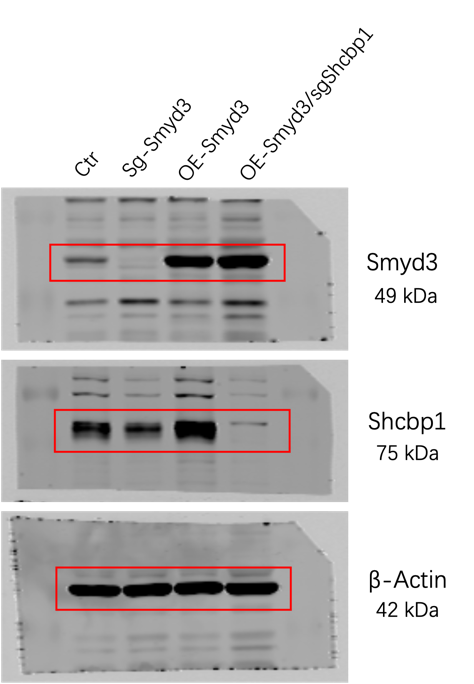
**

**Fig 6f Fig 6g**

**
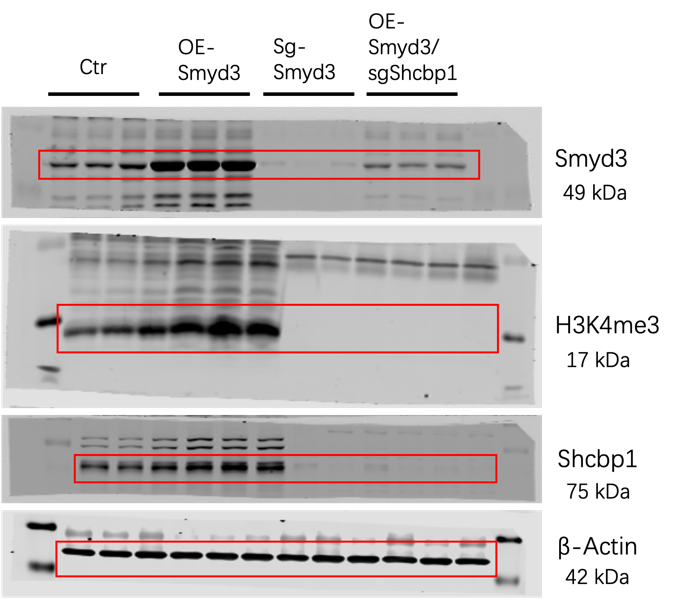

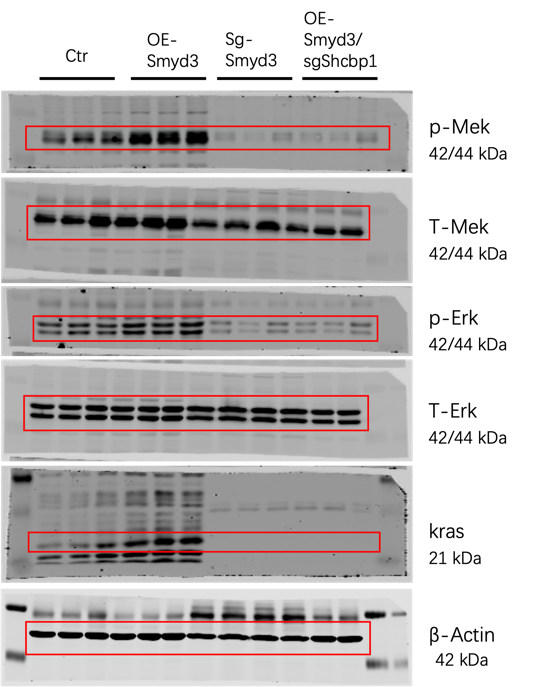
**

**Fig 7k Fig 7l**

**
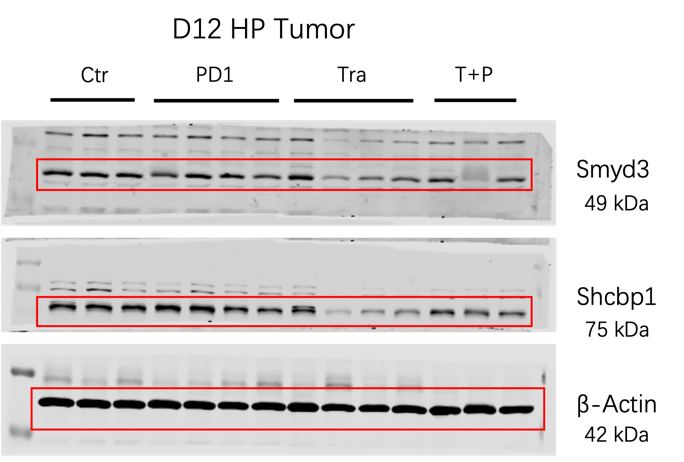

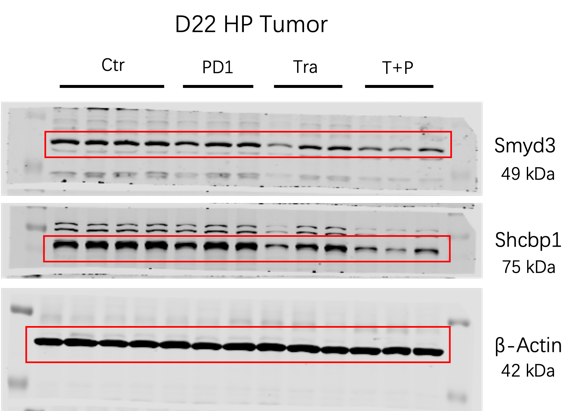
**

**Fig 7m Fig 7n**

**
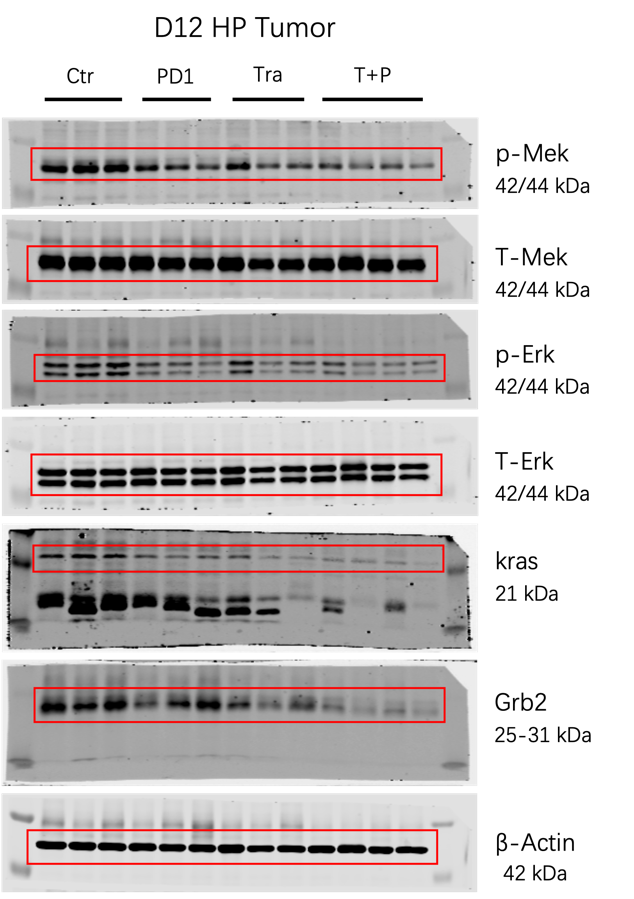

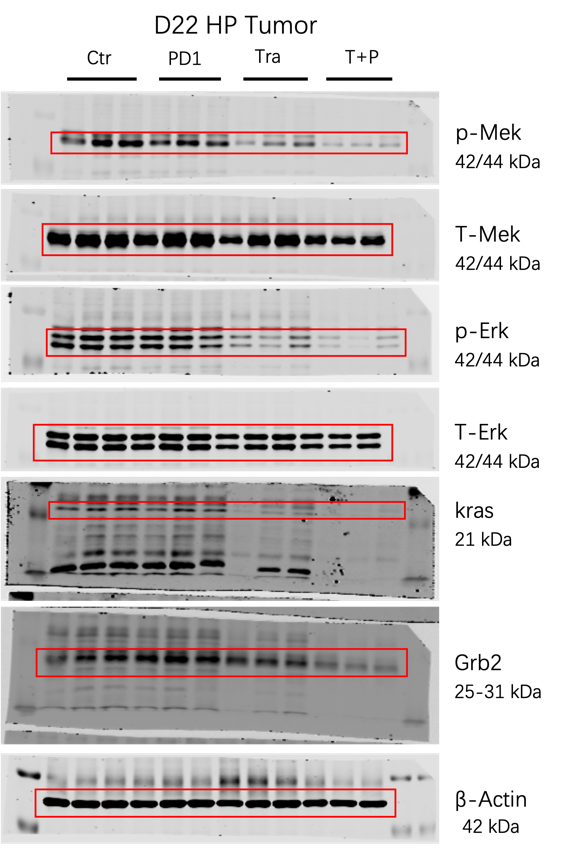
**

**Sup.Fig 2c**

**
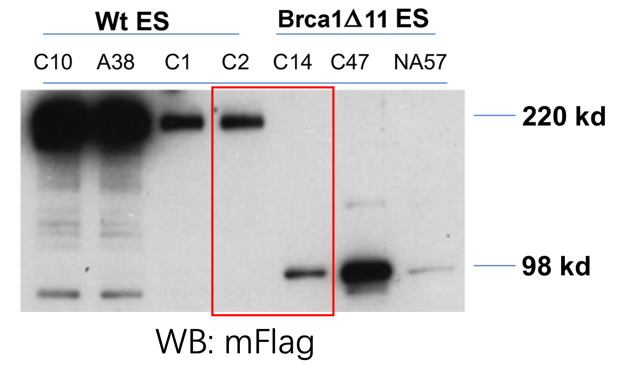

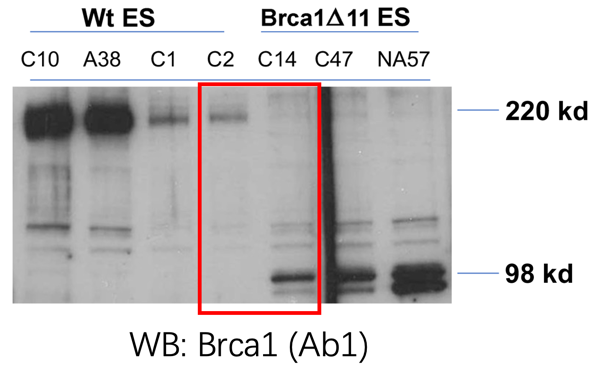
**

**Sup.Fig 8c**

**
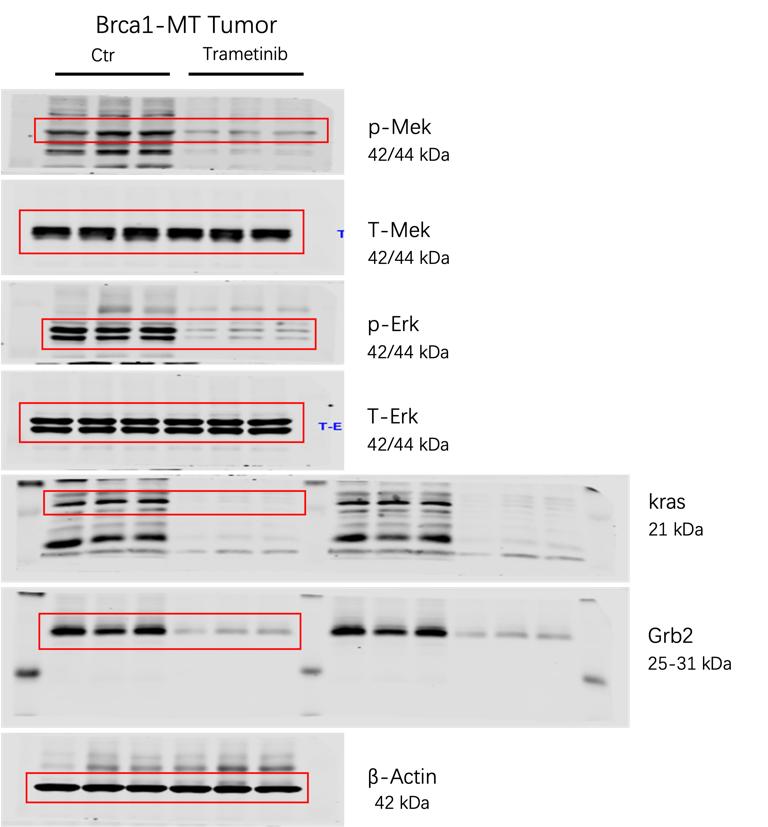
**
